# How to Successfully Navigate Flatland: A Tutorial on the Rheometry of Fluid–Fluid Interfaces

**DOI:** 10.1021/acs.langmuir.6c02098

**Published:** 2026-07-14

**Authors:** Mariana Rodríguez-Hakim, Alexandra Alicke, Javier Tajuelo

**Affiliations:** † Departamento de Física Fundamental, 16757 Universidad Nacional de Educación a Distancia (UNED), Las Rozas de Madrid 28232, Spain; ‡ Department of Mechanical Engineering, Eindhoven University of Technology, P.O. Box 513, 5600 MB Eindhoven, The Netherlands; ¶ Departamento de Física Interdisciplinar, Universidad Nacional de Educación a Distancia (UNED), Las Rozas de Madrid 28232, Spain

## Abstract

Interfacial rheology seeks to understand how fluid–fluid interfaces flow and deform under applied stresses, yet experimental studies are often complicated by the coupled effects of thermodynamics, mechanics, and transport phenomena. This Tutorial provides a practical roadmap for navigating this complexity. We discuss the physical origins of thermodynamic (interfacial tension) and mechanical (rheology) surface stresses and their coupling via the interfacial momentum balance equation, define material functions as the parameters that quantify the rheological properties of a sample, and explain how these are experimentally obtained. Using dimensional analysis, we explain how thermodynamic and mechanical contributions are decoupled through isotherm measurements and how numerical flow field-based data analysis schemes are used to separate bulk and interfacial hydrodynamic stresses. The available instrumentation for shear and dilatational rheometry is reviewed, with emphasis on the importance of kinematically pure deformations and the limitations of mixed-flow techniques. Practical guidelines are provided to help researchers design meaningful experiments, minimize measurement artifacts, and obtain reproducible and physically meaningful data.

## Graphical abstract

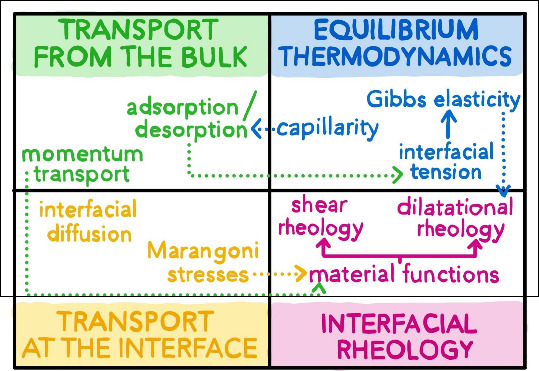

## 1 Introduction

The satirical novel *Flatland*, published by E. Abbott in 1884 and popular among Physics, Mathematics, and Computer Science students, describes a two-dimensional (2D) world populated by lines, triangles, and other planar shapes.^1^ The protagonist, *A Square*, is visited by a sphere that takes it to our more familiar 3D world. This experience makes *A Square* challenge the established mindset of Flatland’s inhabitants about the existence of higher dimensions (with no success). In this story, Flatland is an isolated world: its population lives in their 2D universe with no influence from higher dimensions (except for the sphere’s visit, once every millennium). The interfacial systems that we find and investigate in our real world can also be considered 2D systems, given their very small thickness. Their characterization and, therefore, our life as *Interface Scientists*, would be rather easy if interfaces in the real world were isolated from higher dimensions, as Flatland in Abbot’s novel (in this Tutorial, we will take the liberty to compare Abbot’s novel to the real systems that we explore in the lab by referring to the interface as *Flatland*, and to the species located at the interface as *Flatlanders* or *Flatland inhabitants*).

In practice, simplifying a fluid–fluid (gas–liquid or liquid–liquid) interface as an isolated 2D system is acceptable only in very special and infrequent circumstances, due to the coupling between (i) thermodynamic contributions related to changes in *inhabitant population density* and temperature at the interface, (ii) mechanical (or rheological) effects that depend on how inhabitants respond to Flatland being deformed, and (iii) transport phenomena arising when Flatlanders move around, migrate to/from the bulk, and react to flows in the 3D world.^2–5^ The level of complexity that we must adopt depends on what needs to be analyzed: How is Flatland deformed? Who are its inhabitants? How many are there? Can they travel to the 3D world?

There is no single, pre-established route that the experimentalist can easily follow, as different techniques are developed for, and therefore suited for, interrogating different properties. Each experimental technique provides information specific to the flow or deformation that has been applied, and the subsequent data analysis relies on a series of approximations and assumptions. One of the fundamental skills of a researcher in this field is the ability to determine the most suitable experimental and analytical methods for the application at hand and to interpret the results within their inherent constraints and assumptions.

This “trinity of couplings”—thermodynamics, rheology, and transport—makes interfacial science a challenging discipline. Our goal is to provide general guidelines that can help the reader successfully navigate the world of Flatland. This work is *not* a review article but rather a broad-scoped, practical guide for experimental researchers in interfacial soft materials and rheology. We hope this Tutorial helps its readers design and conduct meaningful experiments and present results with rigor and scientific correctness.

## 2 Where Does the Complexity Come From?

In this section we discuss the different physical phenomena that are found when exploring the properties of an interface and how these phenomena are coupled. Dividing fluid–fluid interfaces into three categories will help us describe their main properties: (1) *clean* interfaces (an empty Flatland), (2) *simple* interfaces (a populated Flatland whose properties only depend on the density and temperature of its inhabitants, i.e., an interface without a rheological response), and (3) *complex* interfaces (in which Flatlanders interact with each other, forming microstructures that render the interfaces resistant to being deformed).^2,6^ Keep these three types of interfaces in mind, as we will refer to them very often throughout the Tutorial.

A molecule in a liquid, surrounded by others in all directions, is under the attraction of all its neighbors and does not show any tendency to move in one particular direction. However, the attraction forces on a molecule at an interface (e.g., a H_2_O molecule at an air–water interface) are unbalanced, yielding a net force toward the aqueous bulk: The molecules are more comfortable in the liquid, surrounded by their similar ones, than at the interface, and they try to leave such energetically unfavorable location.^7,8^ If we want to increase the surface area of an interface, we need to push molecules from the comfort of the bulk toward the interface by performing a given amount of work, similar to the work needed to bring a given mass from the ground-floor to the more energetically unfavorable fifth floor. The **interfacial tension** represents the work that we must perform to increase the surface area of the interface by one unit, and has units of energy per area (J/m^2^).^4,7,9–11^ This is dimensionally equivalent to a force per unit length, and the interfacial tension is usually expressed in mN/m. The physical interpretation of the interfacial tension as energy per area or force per length is intuitive if we perform the thought experiment in Figure 1a. The interfacial tension between phases α and β—water and air, for instance—at temperature *T* is $\sigma_{\alpha \beta }^{0}\left(T\right)$ , where the superscript “0” indicates a clean interface. A *clean* interface (an empty Flatland) can be completely characterized by the interfacial tension, a scalar quantity.^2,5,7,8,12–14^

**Figure 1:**
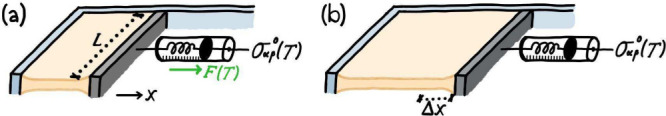
(a) A thin liquid film is confined in a rectangular frame with three fixed sides and a fourth movable side, of length *L*, which is held in place by a dynamometer. The film has two (top and bottom) **clean** air–liquid interfaces. The tendency of the molecules at the interface to leave this unfavorable location manifests as a non-zero reading in the dynamometer, which must be pulled toward the right with a force *F*(*T*) to prevent the film from decreasing its surface area. Thus, the interfacial tension is $\sigma_{\alpha \beta }^{0}\left(T\right)$ = F(T)/(2L) (the factor of 2 accounts for the two interfaces). (b) If we pull the dynamometer very slowly, increasing the film area (and assuming no friction), the force reading remains constant. Moving the barrier by Δ*x* requires a work of W(T) = $2L\sigma_{\alpha \beta }^{0}\left(T\right)\Delta x$ , where the work per unit area increased is $\sigma_{\alpha \beta }^{0}\left(T\right)$ .

### 2.1 Equilibrium Thermodynamics

Now that we have a clearer understanding of what the interfacial tension is, let us discuss the concept of thermodynamic equilibrium, for which we will consider a simple interface. A system is at thermodynamic equilibrium when the net matter and energy flows are zero.^11,15^ Intuitively, we can picture thermodynamic equilibrium as a system whose properties will not change without an external perturbation. Constraining ourselves to interfaces between fluids, thermodynamic equilibrium requires the following:^11,12,15^ (i) The temperature is constant, uniform, and equal to that of the bulk phases. (ii) The concentration of species at the interface is constant or, more precisely, the chemical potential at the interface and the bulk phases is equal. In Flatland, we would say that not only the total population but also the population density in every region must remain constant. (iii) All the forces are balanced, which means that, for flat interfaces, the pressure at the interface and the bulk phases is equal. Flatland’s inhabitants would feel some sort of pressure, but they would not feel a net force in any direction, similar to ourselves under atmospheric pressure.

Let’s go back to our idealized experiment, in which we now have an insoluble surfactant forming a simple interface at constant temperature *T* = *T*_1_ and surfactant concentration Γ = Γ_1_, as shown in Figure 2a. If our interface is at thermodynamic equilibrium, its state is defined by a set of physical quantities called *state variables*, which are mathematically related through *state functions*.^9,11,15,16^ The interfacial tension, σ_*αβ*_(*T*, Γ), is a function of *T* and Γ, these three quantities being state variables.^16^ Thus, the interfacial tension will change if Γ and/or *T* are varied: σ_*αβ*_(*T*_1_, Γ_1_) ≠ σ_*αβ*_(*T*_2_, Γ_2_) (Figure 2b). In general, $\sigma_{\alpha \beta }\left(T_{1},\Gamma_{1}\right)$ < $\sigma_{\alpha \beta }^{0}\left(T_{1}\right)$ ; i.e., the presence of a surfactant decreases the interfacial tension with respect to the corresponding clean interface.^2,7^ The surface pressure is defined as the difference between these two quantities:^6,8,16,17^

1 $$\Pi_{\alpha \beta }\left(T,\Gamma \right)=\sigma_{\alpha \beta }^{0}\left(T\right)-\sigma_{\alpha \beta }\left(T,\Gamma \right)$$

where Π_*αβ*_ can be thought of as a negative interfacial tension, or a *compressive* force per unit length, much like a 2D pressure.^16^

**Figure 2:**
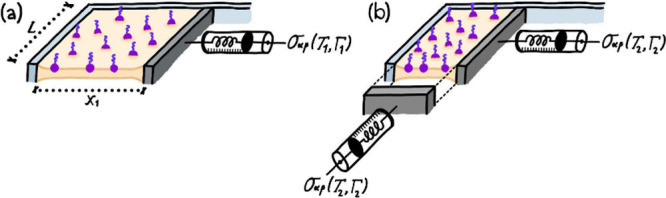
(a) The interfacial tension of a **simple** interface at *T* = *T*_1_ and Γ = Γ_1_ is σ_*αβ*_(*T*_1_, Γ_1_). (b) We compress the interface and change the temperature of the system, so that *T*_2_ ≠ *T*_1_ and Γ_2_ > Γ_1_. If the interface is at equilibrium, the reading of the dynamometer allows us to determine the interfacial tension at this new state, σ_*αβ*_(*T*_2_, Γ_2_), which, in general, differs from σ_*αβ*_(*T*_1_, Γ_1_). The interfacial tension is a scalar quantity: the value of σ_*αβ*_(*T*, Γ) measured by a second dynamometer placed in a different direction is exactly the same.

A fundamental property of state functions is that they do not depend on *how* the interface has reached equilibrium. In our experiment, the interfacial tension reading does not depend on how we have made the interface evolve initially to reach the equilibrium state (*T*_1_, Γ_1_), and later from that state to a new equilibrium state (*T*_2_, Γ_2_). The interfacial tension of a simple interface at a state of thermodynamic equilibrium, defined by a given pair (*T*, Γ), is unique.^18^ Another important property of simple interfaces at thermodynamic equilibrium is that σ_*αβ*_(*T*, Γ) is a scalar quantity: direction does not matter, so the interfacial tension measured by probes oriented along different directions (see Figure 2b) is exactly the same.

Now imagine that we characterize the interface at different equilibrium thermodynamic states, mapping the (*T*, Γ) phase space, and we plot the results for a given temperature, *T*_*i*_, obtaining the curve $\sigma_{\alpha \beta }{\left(\Gamma \right)}_{T_{i}}$ (or the equivalent surface pressure curve, $\Pi_{\alpha \beta }{\left(\Gamma \right)}_{T_{i}}$ ). This curve, represented in Figure 6b, is what we call an *isotherm* and will be discussed in detail in Sections 3.4 and 4.1.2. For now, let’s conclude our discussion by bringing up the paradox that we inevitably face when exploring the thermodynamics of interfaces: If equilibrium is time-independent, but our experiments consist in processes such as compression or heating that develop over time, how can we measure the thermodynamics of an interface without taking into account these effects? To solve this issue we have no option but to make the time-dependent processes so slow that the interface is, at any time, very close to the equilibrium state that corresponds to the instantaneous value of the pair (*T*, Γ). We refer to this kind of processes as *quasi-static*, which consists of a collection of *quasi-equilibrium* states; a good example is the isotherm that results from a quasi-static compression of the interface in Figure 6b.

***Postulate***: Clean and simple interfaces are fully described by a scalar quantity, the interfacial tension, σ_*αβ*_. For clean interfaces, σ_*αβ*_ is a function of temperature— $\sigma_{\alpha \beta }^{0}\left(T\right)$ —whereas for simple interfaces populated by surface active agents, σ_*αβ*_ is a function of *T* and surface concentration—σ_*αβ*_(*T*, Γ). Since σ_*αβ*_(*T*, Γ) is a *state variable*, it does **not** depend on how the interface has been deformed, heated, or cooled in the past.

### 2.2 Transport to/from the Bulk

We can classify the coupling between an interface and the adjacent bulk phases into two categories: **mass-transport** and **momentum-transport**. These concepts can be understood from a very intuitive perspective as the capability of the interface *inhabitants* to travel to/from the interface from/to the bulk (adsorption/desorption) and the coupling between the motion of the interface *inhabitants* and that of the bulk phases, respectively.

### 2.3 Transport at the Interface

This phenomenon can be seen as the motion of Flatland’s population within their home world, where we find driving forces of different nature. **Diffusion** is originated by gradients in concentration, and is equivalent to the tendency of Flatlanders to be more comfortable by occupying the available space in a more homogeneous manner.^19,20^ **Convection** is a more collective motion of the interface inhabitants driven by internal or external forces.^19,20^ For instance, imagine that we, as 3D gods, tilt a planar Flatland so that it is not perpendicular to gravity. Driven by their weight, Flatlanders would migrate toward lower lands. **Marangoni stresses** appear when surface gradients in the interfacial tension are present (i.e., ∇_s_σ_*αβ*_).^19–21^ To differentiate Marangoni stresses from diffusion, imagine that all Flatlanders are homogeneously distributed in the available land, but a gradient in *T* makes some regions too warm to live comfortably in. In that scenario, Flatlanders would move against diffusion, decreasing the population density in the warmer regions. Several mechanisms apart from gradients in *T* can induce gradients in σ_*αβ*_ leading to Marangoni stresses, for instance gradients in Γ ^22–26^ or a non-homogeneous bulk composition.^27–30^

### 2.4 Rheology

Having a rheological or mechanical response is what makes an interface *complex*, and the main goal of this Tutorial is to provide valuable insights into how to differentiate the rheological response of an interface from those discussed previously (thermodynamics, transport to/from the bulk, and transport at the interface).

***Postulate***: Unlike clean and simple interfaces, complex interfaces exhibit two key features:
  (1) Their behavior depends on *how* they are deformed.
  (2) The interfacial tension σ_*αβ*_, is not sufficient to describe their behavior. Instead, we need a *tensorial* quantity that we refer to as the *interfacial stress*, **σ** ^s^.

**Interfacial rheology** arises only when a structure is present at the interface, which can be imparted by different species such as particles, polymers, and proteins that laterally interact, forming microstructural networks that render the interfaces resistant to deformation.^2,6,8,31^ Let us consider a simplified version of a complex interface where Flatlanders have springs instead of arms, they are homogeneously occupying all the available land, and they are holding their neighbors’ hands. If we expand or compress Flatland and, therefore, the structure formed by its inhabitants, our intuition says that we would find some sort of elastic-like resistance. Far from being a mere caricature with no resemblance to reality, this kind of mechanical response is actually seen in real complex interfaces.

To describe the forces in a complex interface and account for both the thermodynamic and the mechanical contributions (including the directional dependency of the latter), we need a *tensorial* quantity, as illustrated in Figure 3. This tensorial quantity is known as the interfacial stress tensor, **σ** ^s^,^3–6,17,32,33^ which is defined as^3–6,17,32,33^

2 $$\sigma^{s}=\sigma_{\alpha \beta }\left(T,\Gamma \right)I_{s}+\tau^{s}$$

where **σ** ^s^ and **τ** ^s^ are symmetric, 2D tensors. Note the resemblance of eq 2 with the definition of the 3D stress tensor,^19,20^ **σ** = −*P* ***I*** + **τ**, with σ_*αβ*_(*T*, Γ) playing the role of a (negative) hydrostatic pressure, *P*. The scalar σ_*αβ*_(*T*, Γ) is multiplying ***I***_s_, the surface identity tensor, defined as ***I***_s_ = ***I*** – ***nn***, where ***I*** is the identity tensor and ***n*** is the unit normal vector that defines the interface orientation. Multiplication with ***I***_s_ tells us that the thermodynamic contribution is isotropic along the interface. The second term on the right-hand side is **τ** ^s^, the interfacial extra stress tensor, which represents the mechanics, or rheology, of the interface. Unlike the state variable σ_*αβ*_, **τ** ^s^ is *path-dependent*, meaning that its components depend on *how* the interface is deformed (e.g., shear or dilatation, see Section 3.5) and the exact trajectory that we take when going from an initial to a final state.^3,4,34,35^

***Postulate***: The *extra stress tensor*, **τ** ^s^, represents the purely mechanical (i.e., rheological) response of the interface when deformed:
  (1) For clean and simple interfaces, **τ** ^s^ = 0.
  (2) For complex interfaces, **τ** ^s^ ≠ 0.

**Figure 3:**
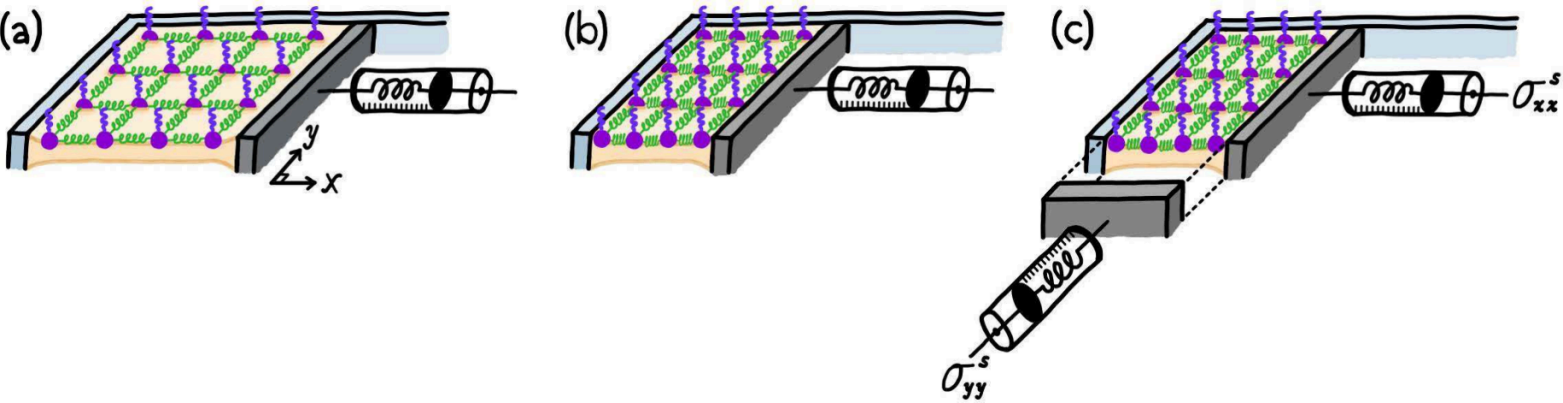
(a) In our idealized experiment, an isothermal **complex** interface is populated with a compound arranged in a rectangular lattice, where neighbors in the *x* and *y* directions are connected by springs. The force measured by the dynamometer is not equal to σ_*αβ*_ because, in principle, we do not know to what extent the springs are stretched along the *x* axis. The reading is the interfacial tension **plus** a contribution due to the springs’ deformation: thermodynamics + mechanics. (b) Both the thermodynamic and the mechanical contributions will change when the interface is compressed, and the dynamometer will return a different reading. More importantly, since the deformation of the springs along the *x* and *y* directions is different, (c) a dynamometer placed along the *y* axis yields a different result, $\sigma_{xx}^{s}$ ≠ $\sigma_{yy}^{s}$ .

For a planar interface within the *x*–*y* plane (***n*** = ***e***_*z*_), the interfacial stress tensor reads

3 $$\sigma^{s}=\left(\begin{array}{ll} \sigma_{xx}^{s} & \sigma_{xy}^{s}\\ \sigma_{yx}^{s} & \sigma_{yy}^{s} \end{array}\right)=\left(\begin{array}{cc} \sigma_{\alpha \beta }\left(T,\Gamma \right) & 0\\ 0 & \sigma_{\alpha \beta }\left(T,\Gamma \right) \end{array}\right)+\left(\begin{array}{ll} \tau_{xx}^{s} & \tau_{xy}^{s}\\ \tau_{yx}^{s} & \tau_{yy}^{s} \end{array}\right)$$

where the first and second subscript indices represent the orientation of our control surface and the direction of the measured force, respectively.^19,20,33^ We will analyze this expression in more detail in Section 3.5. For now, notice that all the stresses lie *tangent* to the interface.^3^ Although interfaces can also exhibit *normal* stresses, such as bending or torsion, these are not discussed here.^11,36,37^

### 2.5 Unraveling Where the Complexity Comes From: The Interfacial Stress Balance Equation

We have seen, in rather qualitative broad strokes, that thermodynamics, transport, and rheology all affect life in Flatland. We must now approach the problem in a more mathematical way (for a thorough discussion, see Venerus and Öttinger,^5^ Slattery et al.,^33^ Sagis,^38^ and Jaensson et al.^3^). Consider the interface as a 2D surface dividing two bulk fluids.^3,5,11,33^ Imagine an arbitrarily small piece of the interface (an *interface element*), with one side in contact with bulk fluid 1 and the other with bulk fluid 2. The dynamics of the interface element is dictated by the external forces applied on it, divided into three categories: (i) forces applied by fluid 1, (ii) forces applied by fluid 2, and (iii) forces applied by the outer region of the interface on the contour of the interface element.^3,19,20^ The addition of these forces, along with other assumptions, yields the interfacial momentum balance equation:^5,33,38^

4 $$\left(\sigma^{1}-\sigma^{2}\right)\cdot n=\nabla_{s}\sigma_{\alpha \beta }-\sigma_{\alpha \beta }\left(\nabla_{s}\cdot n\right)n+\nabla_{s}\cdot \tau^{s}$$

also known as the interfacial stress balance, where ***n*** points toward phase 2 as in Jaensson et al.^3^ On the left, **σ** ^1^ and **σ** ^2^ are the stress tensors of bulk fluids 1 and 2 (i.e., forces applied by fluids 1 and 2 on the interface). If (**σ** ^1^ – **σ** ^2^)· ***n*** ≠ 0, the interface can dissipate or store energy, leading to a discontinuity in the stresses between the two adjacent fluids.

On the right-hand side are all the forces that act along the interface. The surface gradient operator, ∇_s_, describes how a quantity varies along the interface plane.^3,5,33^ The first term, ∇_s_σ_*αβ*_, represents the Marangoni stresses mentioned in Section 2.3. When Flatlanders move along the interface, they must overcome the drag from the adjacent bulk fluids. Until now we have pictured only planar Flatlands, but interfaces can exhibit (and frequently do) curvature. The second term, σ_*αβ*_(∇_s_· ***n***) ***n***, is the capillary contribution (the Laplace pressure in drops or bubbles is precisely this term^5,7,20^ ), where ∇_s_· ***n*** equals half the mean curvature.^5,19,20^ For the remainder of the text, we will mostly constrain ourselves to examining planar Flatlands, since (i) the fundamental physical phenomena can be easily generalized to include curvature effects and (ii) most of the experimental setups that we focus on in this Tutorial operate on planar interfaces. The third term, ∇_s_·**τ** ^s^, is the rheological stress.^2,3,6,8^ For a better understanding of this term, imagine the unrealistic but illustrative case of an infinitely rigid interface. If bulk fluid 1 flows in such a way that it applies a shear stress on the interface (**σ** ^1^· ***n*** ≠ 0), the interface would tend to deform. However, if the interface were infinitely rigid, it would not deform at all, and it would subsequently not transfer any stress to bulk fluid 2, which would remain at rest (**σ** ^2^· ***n*** = 0). Therefore, (**σ** ^1^ – **σ** ^2^)· ***n*** ≠ 0, being the origin of such stress discontinuity in the rheological properties of the interface. Figure 4 summarizes this section, highlighting examples of couplings that are commonly encountered in experimental scenarios, and captures where the complexity comes from: the coupling between thermodynamics, transport, and rheology.

**Figure 4:**
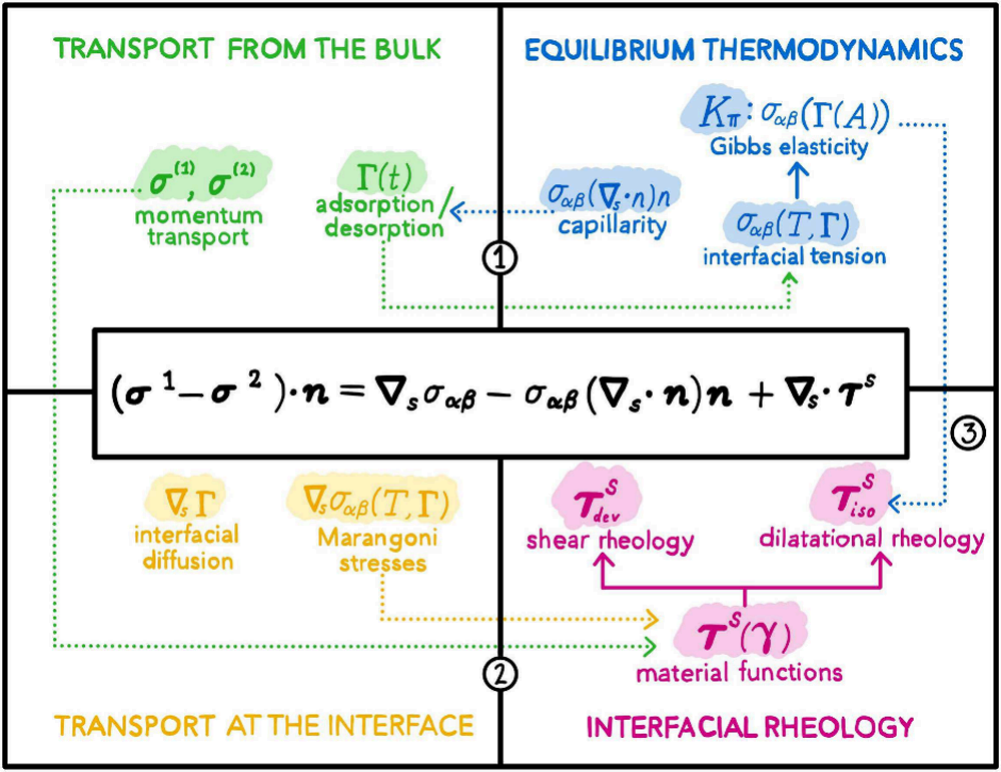
The coupling between thermodynamics, transport, and rheology is captured in the stress balance equation. Examples of different couplings are shown by the dotted arrows: (1) A non-zero interface curvature gives rise to capillary stresses and enhances the adsorption rate of soluble species from the bulk,^39,40^ driving time-dependent changes in Γ that modify σ_*αβ*_. (2) Marangoni and bulk viscous stresses must be decoupled from the experimentally measured stress to obtain true material functions that exclusively reflect the interface’s rheological properties (Sections 3.1, 3.5.1, 4.2.3, and 4.3.3). (3) The Gibbs elasticity—a measure of the interface’s thermodynamic compressibility—is often convoluted with or mistaken for the true rheological elasticity, yielding an *apparent* dilatational response (Sections 3.1 and 3.4.2).

## 3 What Do You Want to Measure?

As experimentalists, our work comprises acquiring data utilizing a properly selected technique and in analyzing and interpreting these data, accounting for all of the phenomena affecting our experiment. If the technique was well selected, the phenomenon we are interested in should be the most relevant in our measurements, but even then, we must account for any possible couplings that can lead us to incorrectly analyze our data.

### 3.1 Constitutive Models: True and Effective Material Functions

**Material functions** are the physical parameters that describe the rheological behavior of our sample, and their experimental determination is the ultimate goal of any interfacial rheometry technique. Material functions prescribe a quantitative relationship between the *rheological stress* and *deformation* of an interface. Different material functions can be defined, such as the interfacial shear viscosity, η_s_, the interfacial shear modulus, *G*_s_, the interfacial dilatational modulus, *K*_s_, among others listed in the Society of Rheology’s official nomenclature.^41^ The application we are interested in tells us which material functions we must explore and, accordingly, the most suitable experimental technique.

The data one collects from a rheological experiment are the stress and the strain or, more precisely, at least one of the components of the stress and strain tensors at a given location (remember that the stress tensor, **σ** ^s^, is a combination of thermodynamics and mechanics).^31,35^ Let us assume for now that the decoupling of thermodynamics and mechanics is feasible in our experiment, i.e., we are able to extract **τ** ^s^ from the experimentally measured **σ** ^s^. In such cases, we are in the position to find the material functions, but first, we need a *constitutive model* that relates our data (stress and strain) to the proper material functions.^8,13,42^

A constitutive model is just a mathematical relationship which, as any other physical model, provides a simplified yet accurate representation of nature written in the language of mathematics. In Interfacial Rheology, constitutive models have the following general form:

5 $$\tau^{s}=f\left(\mathrm{material}\;\mathrm{functions},\gamma^{s},{\mathrm{\gamma ̇}}^{s}\right)$$

where **γ** ^s^ is some form of the interfacial strain tensor. One can define different strain tensors,^3,8,13,42,43^ but to understand the concept of a constitutive model all we need to know is that **γ** ^s^ contains information on the deformation of an interface element and is related to its solid-like (elastic) properties.^3,8,13,32,42–44^ Similarly, **γ̇** ^s^ is some form of the interfacial rate-of-strain tensor^3,8,45^ (d**γ** ^s^/d*t*) and is related to the fluid-like (viscous) response.^19,20,33,45,46^

There are several constitutive models in Interfacial Rheology with different levels of complexity in the function *f* that resemble their better known counterparts in bulk rheology. The Boussinesq–Scriven model, the 2D analogue of Newtonian fluids, is typically used to describe the **viscous** response, where a linear relationship is established between **γ̇** ^s^ and **τ** ^s^.^46^ Although nonlinear models in strain are often required to capture the elastic properties under larger deformations, the linear Boussinesq–Scriven model^46^ can adequately describe the viscous behavior of an interface even under large strain rates, analogously to the linear relationships in bulk fluid mechanics.

To capture the **elastic** response, different models have been developed. For small deformations, elastic interfaces may be properly described by the interfacial analogue of Hooke’s law, where **γ** ^s^ and **τ** ^s^ are linearly coupled.^8^ Adding this model to the Boussinesq–Scriven equation leads to the complex Boussinesq–Scriven model, which describes the viscoelastic response under oscillatory flows.^31,47,48^ More sophisticated elastic constitutive models, such as the Hencky or neo-Hookean models, should be selected when operating at larger strains.^3,8,13,42–44^

A properly selected constitutive model, that captures the actual response of our sample under the deformations or stresses applied in our experiments, not only allows us to find the material functions, but also has the capability to predict the sample’s behavior under other circumstances. Thus, material functions can be used as *inputs* in constitutive models to predict the interface’s response under other conditions. For more details on constitutive models, refer to Jaensson et al.,^3^ Verwijlen et al.,^8^ Pepicelli et al.,^13^ and Carrozza et al.^42^

***Postulate***: The outcome of an interfacial rheological experiment is the total interfacial stress, **σ** ^s^, yet the input for the constitutive models is the rheological (extra) stress, **τ** ^s^. When **τ** ^s^ can be extracted from the experimentally measured **σ** ^s^, it can be used in combination with an adequate constitutive model to calculate the relevant interfacial material functions.

Now let’s discuss how to face the non-ideal but frequent cases where the ultimate goal of finding the material functions is not possible. In experiments, we obtain (one or more components of) **σ** ^s^, but constitutive models require **τ** ^s^ as an input. Can we, somehow, obtain **τ** ^s^ from the experimentally measured **σ** ^s^? If the answer is *no*, we must settle for a more or less quantitative correlation between **σ** ^s^ and **γ** ^s^ and avoid any attempt to calculate *true* material functions. We are still able to find useful information on how the measured stress relates to the deformation, but we cannot claim to have characterized the rheological properties of our interface from such experiments. At most, we might define *effective* or *apparent* material functions by applying a constitutive model to the *total* interfacial stress:^14,17^

6 $$\sigma^{s}=f\left(\mathrm{effective}\;\mathrm{material}\;\mathrm{functions},\gamma^{s},{\mathrm{\gamma ̇}}^{s}\right)$$

Notice the differences between eqs 5 and 6.

***Postulate***: If thermodynamics and mechanics cannot be decoupled in an experiment (which is rather frequent), we must settle for our measurement being a combination of both. In this case, we must **not** present results in terms of *true* material functions, as these describe the sample’s mechanical response, while our data also have a thermodynamic contribution. *Effective* material functions, which rely on the total stress **σ** ^s^, should be used instead.

Aside from the coupling between thermodynamics and mechanics, bulk and interfacial transport may also be present and contribute to the measurement of **σ** ^s^ (bulk momentum transport will be discussed in Sections 3.5.1 and 4.2.3). Figure 5 presents a Roadmap to guide the reader in systematically identifying when such couplings arise and what measurements can be conducted for different types of interfaces, ultimately leading to the correct characterization of the material functions (*how* to obtain them will be addressed in Section 4).

**Figure 5:**
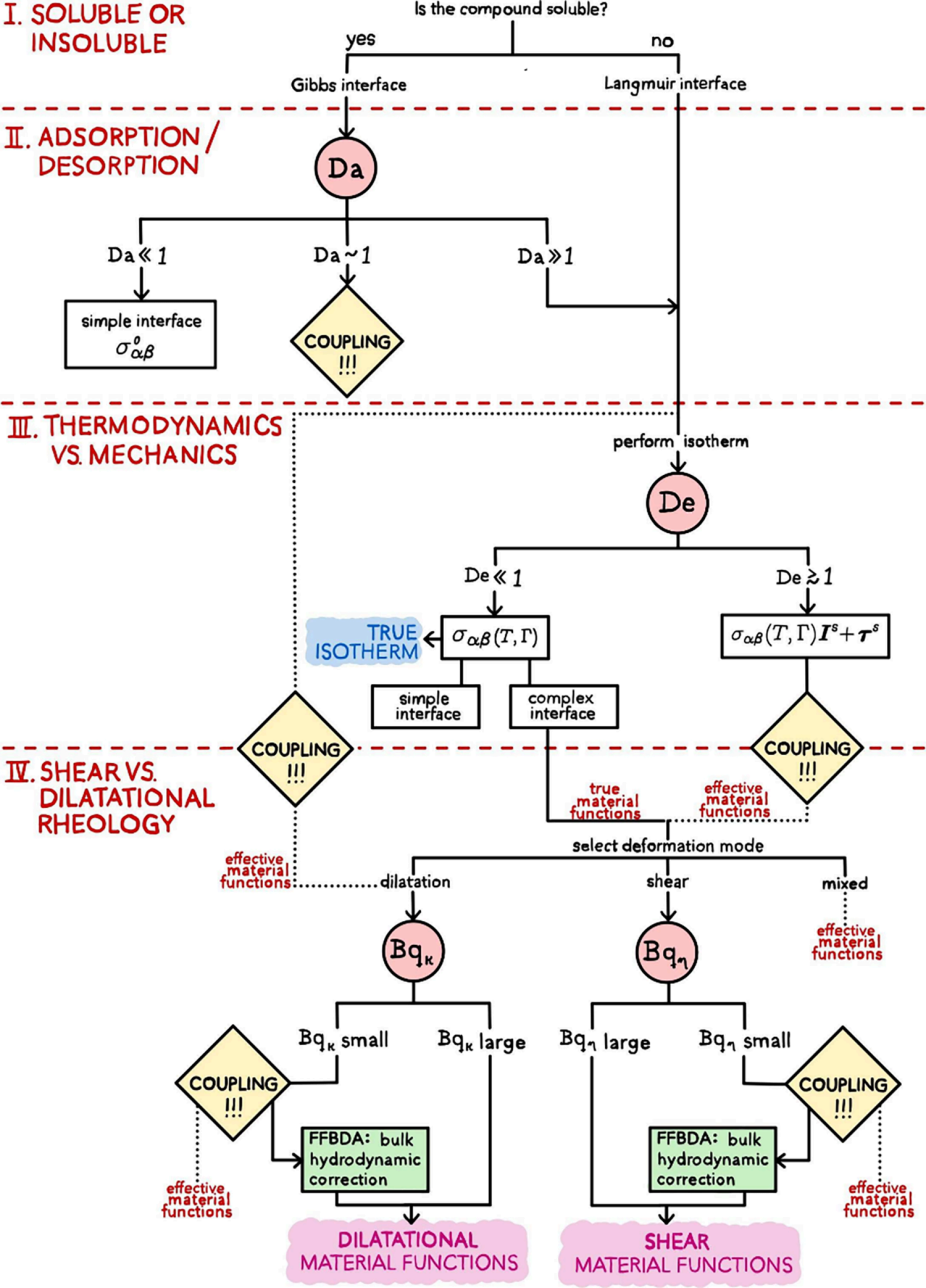
Roadmap for interfacial scientists.

### 3.2 Soluble or Insoluble?

The first step in our Roadmap is to determine the identity of Flatland’s inhabitants. In Langmuir (insoluble) interfaces, these inhabitants are insoluble in both bulk phases; they remain confined to the interface, while momentum is still allowed to visit the 3D world. To create an insoluble monolayer, a known number of Flatlanders, *N*_s_, is deposited at the interface (see Section 4.4.2). Since mass transfer with the bulk is absent and *N*_s_ remains constant, Γ is known and equilibrium thermodynamics can, in principle, be fully decoupled from bulk transport effects (see Section 3.4). In contrast, soluble (Gibbs) interfaces are easier to form, since all we must do is wait for Flatlanders to diffuse through the bulk and adsorb at the interface. However, mass transport between the bulk and the interface must be considered, especially with changes in the surface area or curvature, which can induce adsorption/desorption phenomena.^2,19^ Determining Γ for soluble species is sometimes not possible (particularly for species that readily adsorb or desorb), so σ_*αβ*_(Γ, *T*) is not known and bulk transport, thermodynamic, and mechanical effects remain inherently coupled.

### 3.3 Adsorption/Desorption

#### 3.3.1 The Damköhler Number: Decoupling Mass Transport Effects

Adsorption and desorption (collectively known as sorption) are *kinetic processes*, and there are scenarios where mass transport to/from the bulk can be decoupled from the interfacial response in Gibbs interfaces.^39,49,50^ Whether this is possible depends on their relative time scales. The adsorption and desorption time scales, *t*_ads_ and *t*_des_, can be classified as fast or slow relative to a reference time scale—generally chosen to be the process time scale, *t*_proc_—which describes the duration of our experiment or process of interest. For example, in oscillatory rheological experiments, *t*_proc_ ∼ 1/ω, where ω is the oscillation frequency. We can define a dimensionless Damköhler number (Da) to quantify this ratio of time scales:

7 $${\mathrm{Da}}_{a}=\frac{t_{\mathrm{ads}}}{t_{\mathrm{proc}}},,{\mathrm{Da}}_{d}=\frac{t_{\mathrm{des}}}{t_{\mathrm{proc}}}$$

In the limits of Da ≪ 1 or Da ≫ 1, sorption can be fully decoupled from our experiment: When Da_a_, Da_d_ ≪ 1, an equilibrium Γ will be reached instantaneously. This scenario is usually encountered with small soluble surfactants (e.g., SDS, SLS, Span 20), which are incapable of forming interfacial networks and are considered simple interfaces.^17^ When Da_a_, Da_d_ ≫ 1, sorption will proceed so slowly that our interface effectively behaves as if it were insoluble; thus, we can in principle decouple thermodynamic from mechanical effects to calculate true material functions (see Section 3.1).

The intermediate cases are more complicated. Da_a_, Da_d_ ≈ 1 indicates that the sorption and process time scales are similar, so sorption will be relevant throughout our experiment. Cases where Da_a_ ≪ 1 and Da_d_ ≫ 1 (or vice versa) can also get us into trouble, since a disparity in time scales can induce hysteretic effects. In both of these scenarios Γ is unknown, and we cannot obtain true material functions because we cannot attribute how much of the variations in **σ** ^s^ are due to thermodynamics or mechanics. However, we can still learn a lot about our interfaces by calculating *effective* material functions (see Section 3.1), and documenting the observed behavior in response to a well-designed experiment that represents, to the extent possible, an accurate reflection of the *real* phenomenon or process we are examining.

### 3.4 Thermodynamics vs Mechanics

When thermodynamics and mechanics can be decoupled, true isotherms (purely thermodynamic response) and true material functions (purely mechanical response) can be measured.

#### 3.4.1 The Deborah Number: When Is a True Isotherm Being Measured?

An isotherm is obtained by slowly compressing a Langmuir interface (i.e., increasing Γ by reducing the surface area, *A*) at constant temperature *T*_*i*_, while measuring the surface stress (practical considerations are discussed in Section 4.1). Although the quantity we are interested in is σ_*αβ*_ (or, equivalently, Π_*αβ*_), the variable that we are actually measuring in our experiments is one of the components of the total surface stress, **σ** ^s^, as shown in Figure 6a.

**Figure 6:**
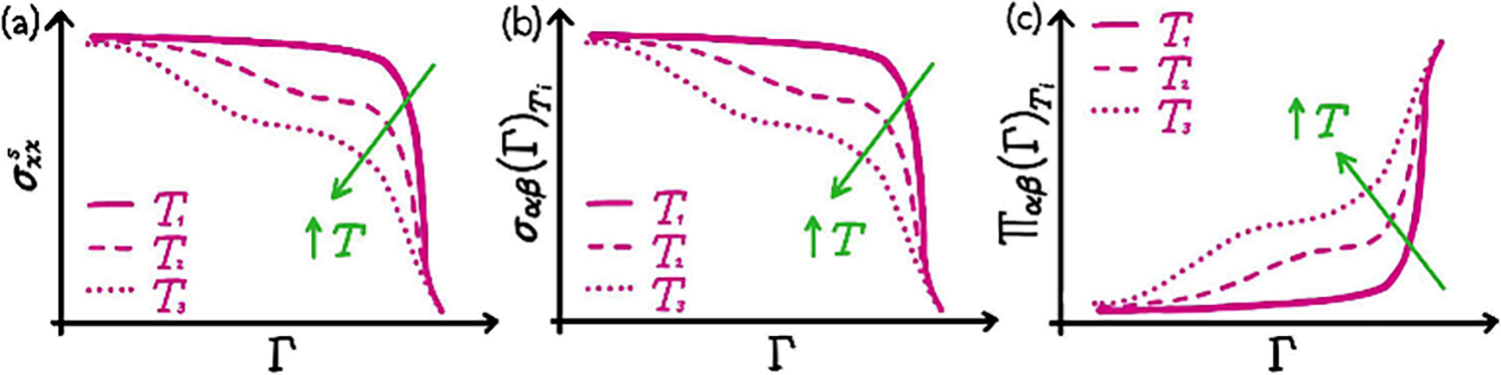
(a) We measure $\sigma_{xx}^{s}$ vs Γ at three different temperatures *T*_*i*_, depicted by the solid, dashed, and dotted lines. (b) An isotherm represents the relationship between two state variables—σ_*αβ*_ and Γ—at fixed *T*_*i*_: $\sigma_{\alpha \beta }{\left(\Gamma \right)}_{T_{i}}$ . (c) Isotherms can also be represented in terms of the surface pressure: $\Pi_{\alpha \beta }{\left(\Gamma \right)}_{T_{i}}$ .

For simple interfaces, isotherms are relatively easy to obtain because there is no rheological response, so the measured stress is equivalent to the interfacial tension: **σ** ^s^ = σ_*αβ*_ ***I***_s_. In such cases, we can directly present our measurements as $\sigma_{\alpha \beta }{\left(\Gamma \right)}_{T_{i}}$ (Figure 6b) and safely claim that we measured an isotherm (a *true* one). More importantly, the thermodynamics of our sample is fully determined: having the isotherm $\sigma_{\alpha \beta }{\left(\Gamma \right)}_{T_{i}}$ in our hands, the value of σ_*αβ*_ can be obtained *for any other experiment* in which *T* = *T*_*i*_ and Γ is known (and lies within the range of our isotherm). Isotherms are extremely useful tools to deconvolute the thermodynamic and rheological contributions in our experiments. To understand why, remember that σ_*αβ*_ is uniquely defined by the pair (T,Γ) (see Section 2.1). Thus, knowing that the interfacial tension is $\sigma_{\alpha \beta }^{*}$ at *T* = T* and Γ = Γ* (i.e., $\sigma_{\alpha \beta }\left(T^{*},\Gamma^{*}\right)$ = $\sigma_{\alpha \beta }^{*}$ ), automatically tells us that $\sigma_{\alpha \beta }=\sigma_{\alpha \beta }^{*}$ *for any other independent experiment* in which *T* = T* and Γ = Γ*.^8,13,51^

***Postulate***: Isotherms represent the relationship between thermodynamic state variables—σ_*αβ*_ vs Γ—at constant *T*, $\sigma_{\alpha \beta }{\left(\Gamma \right)}_{T}$ . Isotherms are useful because they allow us to obtain σ_*αβ*_ at any known Γ and *T*, regardless of the experiment we are performing. Thus, thermodynamics can be decoupled from the stress measured in any other experiment.

For complex interfaces, where **σ** ^*s*^ contains contributions from thermodynamics and mechanics, isotherms are trickier to measure. Care must be taken to minimize the rheological signal, such that **τ** ^s^ ≪ σ_*αβ*_ ***I***_s_ and **σ** ^s^ ≃ σ_*αβ*_ ***I***_s_. In these very frequent cases, the leap from saying *“I measured the surface stress versus concentration”* (Figure 6a) to saying *“I measured the isotherm”* (Figure 6b) is not trivial. Since isotherms must be measured at quasi-equilibrium conditions (i.e., very, very slowly, see Section 2.1), a second dimensionless number, the Deborah number (De),^13^ can help us assess whether such conditions are maintained, where

8 $$\mathrm{De}=\frac{t_{\mathrm{rheo}}}{t_{\mathrm{proc}}}=\frac{\lambda }{t_{\mathrm{proc}}}$$

Here, λ is the viscoelastic relaxation time of the interface and *t*_proc_ is the process time scale (i.e., the reciprocal of the strain rate or oscillation frequency). Details on how to obtain estimates for λ and γ̇ are provided in Pepicelli et al.^13^ For mechanical contributions to be negligible, De ≪ 1. In other words, the compression must be slow enough that all viscoelastic (rheological) stresses have time to dissipate completely (assuming a Maxwell-like model) and for the measured stress to be purely thermodynamic. If De ≥ 1, the measurement will *not* be a true isotherm.^13,14,18,35,52,53^

***Postulate***: In an isotherm, the measured experimental variable is one of the components of the total stress, **σ** ^s^, yet the quantity of interest is the interfacial tension, σ_*αβ*_. To ensure that **σ** ^s^ ≃ σ_*αβ*_ ***I***_s_, experiments must be conducted under quasi-equilibrium conditions (De ≪ 1).

#### 3.4.2 Don’t Be Fooled by the Gibbs Elasticity

Although isotherms can be very useful, we need to make sure we understand exactly what information they convey. A common misconception in interface science is the Gibbs elasticity, *K*_Π_, defined as the change in σ_*αβ*_ with respect to the relative interfacial area, *A*/*A*_0_ (where *A*_0_ and *A* are the initial and final interface areas, i.e., before and after the deformation):^45,54,55^

9 $$K_{\Pi }=\frac{A}{A_{0}}{\left(\frac{\partial \sigma_{\alpha \beta }}{\partial \left(A/A_{0}\right)}\right)}_{T,N_{s}}={\left(\frac{\partial \sigma_{\alpha \beta }}{\partial \ln A}\right)}_{T,N_{s}}$$

Isotherms tell us how σ_*αβ*_ changes with Γ, and since Γ = Γ_0_ *A*_0_/*A* for an insoluble interface, isotherms also tell us how σ_*αβ*_ changes with *A*/*A*_0_. But, why is *K*_Π_ referred to as an elasticity? And, is it related to the rheological elasticity (i.e., solid-like response) of complex interfaces?

To answer this question, let’s abandon Flatland and venture to the more familiar 3D world. Imagine the typical example from a classical thermodynamics course, where *N* moles of an ideal gas at constant temperature *T*_0_ are held inside a piston–cylinder assembly, as shown in Figure 7a. We perform a series of isothermal expansions and compressions by oscillating the piston up and down. If the piston is moved along the +*x* axis, the gas is compressed and its density, *N*/*V*, and pressure, *P*, increase linearly according to the ideal gas law, as shown by the isotherm in Figure 7b. The changes in *P* and the piston displacement Δ*x* (Figure 7c) are characteristic of an elastic solid, where the *stress*, represented by *P*, changes proportionally to the *displacement*. However, we are aware that ideal gases are not elastic solids and that the observed behavior reflects the *compressibility* of the gas, i.e., its tendency to increase (decrease) its density with an increase (decrease) in the pressure.

**Figure 7:**
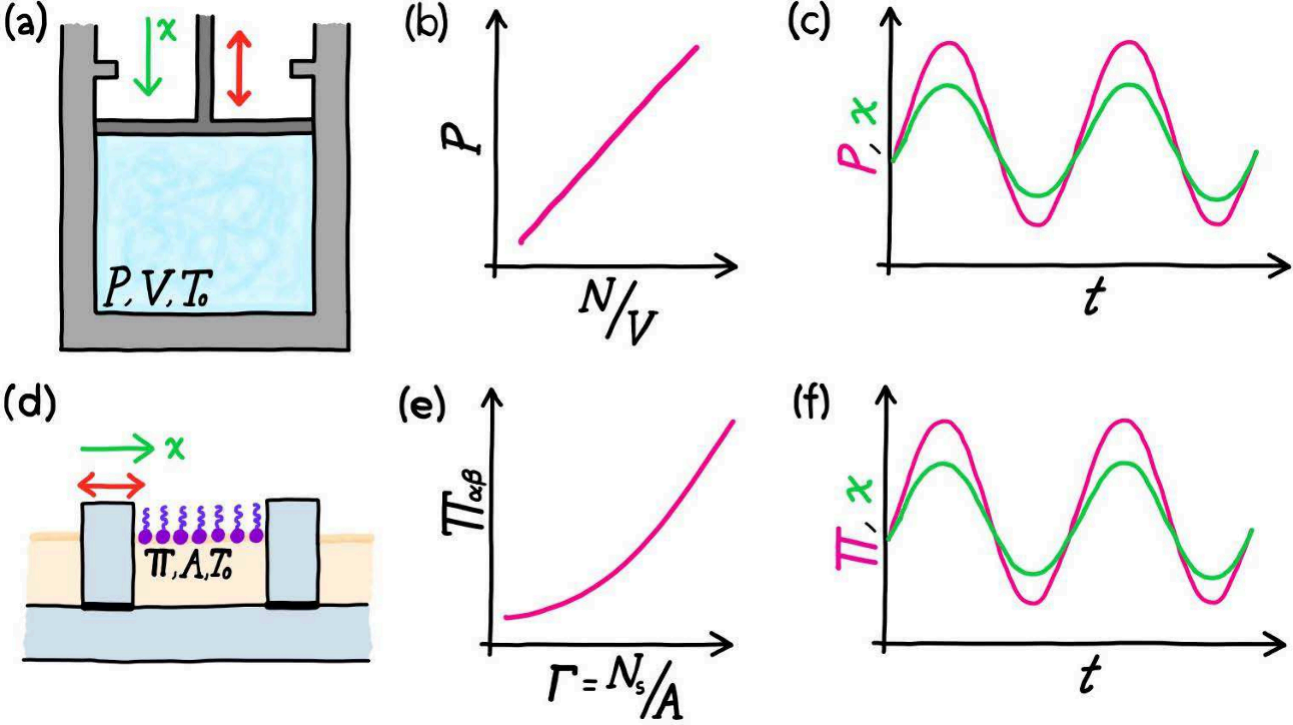
(a) An isothermal ideal gas is held inside a piston–cylinder assembly and the piston is oscillated along the *x* axis. (b) The isotherm shows the relationship between the pressure, *P*, and the volume density, *N*/*V*. (c) The in-phase variation between *P* and the piston displacement, Δ*x*, reflects the thermodynamic compressibility of the gas. (d) By analogy, an insoluble and isothermal simple interface is compressed and expanded by oscillating the left barrier along the *x* axis. (e) The isotherm shows the variation between Π_*αβ*_ and Γ. (f) The in-phase variation between the stress and strain reflects the thermodynamic compressibility of the interface (i.e., Gibbs elasticity) and not a real solid-like elastic behavior.

Let’s now return to Flatland. Consider a simple interface, which is fully described by Π_*αβ*_ (or, equivalently, σ_*αβ*_), the 2D analogue of *P*. Our interface contains a constant number of inhabitants, *N*_s_, is kept at constant *T*_0_, and is subjected to a series of oscillatory expansions and compressions, as shown in Figure 7d. If the barrier is moved along the +*x* axis, the interface is compressed and both its density per unit area, Γ = *N*_s_/*A*, and Π_*αβ*_ increase. We can construct an isotherm which shows the change in Π_*αβ*_ with Γ (Figure 7e) and plot the changes in Π_*αβ*_ and *x* as functions of time (Figure 7f), where we again recognize that *x* ∝ Π_*αβ*_, resembling the response of an elastic solid. This *apparent* elastic response is the Gibbs Elasticity, *K*_Π_.^6,13,14^ Analogously to the 3D case, *K*_Π_ represents the compressibility of the interface—a thermodynamic relationship between pressure and density—and *not* a true solid elastic behavior.^6,13,14^ Thus, *K*_Π_ is *not* a true material function.

***Postulate***: The Gibbs elasticity, *K*_Π_, captures the thermodynamic compressibility (a relationship between Π_*αβ*_ and *A*/*A*_0_) and **does not** reflect a true solid elastic behavior; thus, *K*_Π_ should **never** be confused with the *rheological*, or mechanical, dilatational elasticity.

The danger of this thermodynamic relationship and its unfortunate nomenclature is that oftentimes *K*_Π_ is mistaken for the mechanical elasticity (a true material function) that arises in complex interfaces with a solid-like response. For example, there is abundant literature in which oscillatory deformations are applied to an interface, and the elastic dilatational modulus is defined as the portion of the measured stress that lies in-phase with the strain.^56–60^ Why is this an erroneous interpretation of the experimental data? Remember that our experimental probes measure one of the components of the total surface stress **σ** ^s^, which for complex interfaces contains both thermodynamic and rheological contributions. Thus, if *A*/*A*_0_ changes, a response can be elicited in both σ_*αβ*_ (the Gibbs elasticity) and **τ** ^s^ (the *true* mechanical elasticity); however, only **τ** ^s^ should be considered when calculating the true elastic dilatational modulus, *K*_s_ (see Section 3.1). Equally dangerous are situations arising in simple interfaces, where it can be erroneously affirmed that the interface exhibits a viscoelastic response, but what is really being measured is *K*_Π_.

### 3.5 Shear vs Dilatational Rheology

The final step on our Roadmap is to examine the *rheometry* of complex interfaces, which involves the determination of **τ** ^s^. For our convenience, **τ** ^s^ can be split into two terms,

10 $$\tau^{s}=\left(\begin{array}{cc} \tau_{xx}^{s} & \tau_{xy}^{s}\\ \tau_{yx}^{s} & \tau_{yy}^{s} \end{array}\right)=\overset{\mathrm{isotropic}\;\mathrm{stress,}\;\tau_{\mathrm{iso}}^{s}}{\overset{︷}{\left(\begin{array}{cc} \tau_{\mathrm{iso}}^{s} & 0\\ 0 & \tau_{\mathrm{iso}}^{s} \end{array}\right)}}+\overset{\mathrm{deviatoric}\;\mathrm{stress,}\;\tau_{\mathrm{dev}}^{s}}{\overset{︷}{\left(\begin{array}{cc} \tau_{xx}^{s}-\tau_{\mathrm{iso}}^{s} & \tau_{xy}^{s}\\ \tau_{yx}^{s} & \tau_{yy}^{s}-\tau_{\mathrm{iso}}^{s} \end{array}\right)}}$$

corresponding to the isotropic $\left(\tau_{\mathrm{iso}}^{s}\right)$ and the deviatoric $\left(\tau_{\mathrm{dev}}^{s}\right)$ portions of the extra stress.^61^ Deviatoric extra stresses, also known as shear rheological stresses, are generated when the interface *shape* changes while its surface *area* is kept constant, as shown in Figure 8.^14,31,35,62–64^ Isotropic extra stresses, also known as dilatational rheological stresses, arise when an interface changes its surface *area* while maintaining its *shape* and aspect ratio constant.^12–14,35^ Unlike for bulk materials, where the Poisson ratio sets a bound for the ratio between the shear and dilatational moduli, for 2D interfaces separate measurements are needed for shear and dilatation.^65,66^

**Figure 8:**
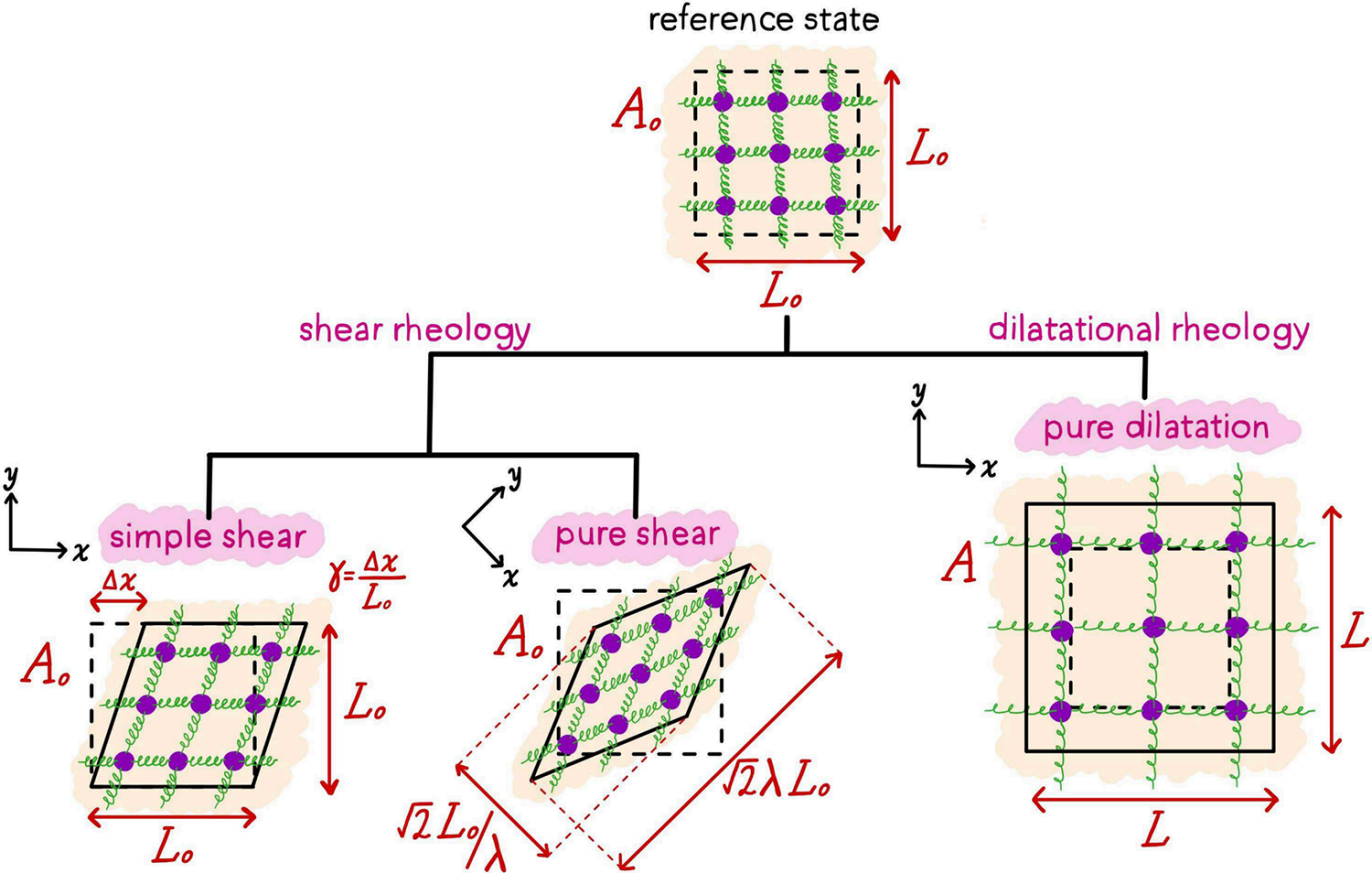
An idealized complex interface, populated by Flatlanders connected via springs, is shown at the top, representing an un-deformed reference state with surface area *A*_0_ and side length *L*_0_. In simple or pure shear, the interface shape changes while its area remains constant; in pure dilatation, the shape of the interface remains constant and its area changes.

A priori, there is no preferred deformation—whether we choose to apply shear or dilatation depends on the application at hand. However, the cleanest data sets are obtained from experiments with *kinematically pure* deformations; that is, when shear and dilatation are applied separately. The deformations must also be *viscometric*, which means that the strain and stress fields can be quantitatively obtained (either analytically or computationally). Analytical solutions are possible when the strain is spatially homogeneous, although non-homogeneous strain fields can also be numerically calculated, as discussed in Sections 4.2.3 and 4.3.3.

***Postulate***: Rheological extra stresses, **τ** ^*s*^, arise whenever an interface exhibits a mechanical resistance against deformation. Extra stresses can be divided into two components:
  (1)
$\tau_{\mathrm{iso}}^{s}$ : Isotropic dilatation (area-changing, shape-preserving)
  (2)
$\tau_{\mathrm{dev}}^{s}$ : Deviatoric shear (shape-changing, area-preserving)
  Usually, it is desirable to probe materials exclusively under shear **or** dilatation, but not under mixed flow fields where shear + dilatation are simultaneously applied.

#### 3.5.1 The Boussinesq Number: Do We Care about What Happens in the Bulk?

Unless the interface is deformed quasi-statically, the motion at the interface will also generate motion in the adjacent bulk phases.^31,47,48,67–70^ Thus, the velocities and stresses at the interface and bulk will be inherently coupled (see Section 2.5), leading to spatially varying strains and stresses. To determine whether bulk hydrodynamics influences our measurements, we define the Boussinesq numbers from eq 4 as the ratio of interfacial to bulk stresses:

11 $${\mathrm{Bq}}_{\eta }=\frac{\tau_{\mathrm{dev}}^{s}}{\sigma^{s}l},,{\mathrm{Bq}}_{\kappa }=\frac{\tau_{\mathrm{iso}}^{s}}{\sigma^{s}l}$$

where τ^s^ and σ^s^ indicate the relevant components of the surface and bulk stress tensors, respectively, and *l* is a length scale. The specific physical variables used in the definitions of τ^s^, σ^s^, and *l* (i.e., characteristic velocity, length, and time scales) must be the relevant ones for our experiment, accounting for its geometry and working principles. More information is provided in Section 4.2.3, but for now, all we need to remember is that if Bq_η_ and Bq_κ_ ≫ 1, bulk hydrodynamics can be safely neglected.^31,32,47,48^ However, if Bq_η_ or Bq_κ_ ≲ 1, the bulk and interface will be coupled. This implies not only that the bulk flow field must be known to solve for the interfacial motion but also that the bulk flow itself depends on the motion of the interface, requiring numerical schemes to determine the bulk and interfacial hydrodynamics simultaneously. Fortunately, many of these numerical routines are already developed for several commercial interfacial shear rheometers and are available as open-source.^47,47,69,71–76^

#### 3.5.2 Shear Rheology

Shear stresses, represented by $\tau_{\mathrm{dev}}^{s}$ , arise when the interface changes shape while maintaining a constant area. To be spatially homogeneous, the surface area must be locally conserved for each interface element. For insoluble and isothermal interfaces, Γ (and, therefore, σ_*αβ*_) remains constant and *changes* in **σ** ^s^ directly correspond to *changes* in $\tau_{\mathrm{dev}}^{s}$ . Thus, a main advantage of shear deformations is that thermodynamics and mechanics can be easily separated in experiments. Constraining ourselves to planar, isothermal interfaces, let’s look at the different ways in which a kinematically pure and viscometric shear stress can be elicited.

##### 3.5.2.1 Simple Shear

In simple shear (Figure 8, left), a unidirectional deformation is applied in the *x*-direction, with a magnitude proportional to the position on the *y*-axis, resulting in a shear strain of γ.^68,77,78^ This deformation is used by most interfacial shear rheometers and all commercial rheometers to date. For linear constitutive models (e.g., the Boussinesq–Scriven model), $\tau_{\mathrm{dev}}^{s}$ for simple shear with reference frame as shown in Figure 8 has the following general form (normal stresses may arise in nonlinear models, such as the Hencky or neo-Hookean models^8,13,42,43^ ):

12 $$\tau_{\mathrm{dev}}^{s}=\left(\begin{array}{cc} 0 & \tau_{xy}^{s}\\ \tau_{yx}^{s} & 0 \end{array}\right)=\left(\begin{array}{cc} 0 & \tau_{xy}^{s}\\ \tau_{xy}^{s} & 0 \end{array}\right)$$

If we now consider **σ** ^s^, as defined in eq 3, including both the thermodynamic and the mechanical contribution from the shear stress (i.e., $\sigma^{s}$ = $\sigma_{\alpha \beta }I_{s}$ + $\tau_{\mathrm{dev}}^{s}$ ), we obtain

13 $$\sigma^{s}=\left(\begin{array}{cc} \sigma_{xx}^{s} & \sigma_{xy}^{s}\\ \sigma_{yx}^{s} & \sigma_{yy}^{s} \end{array}\right)=\left(\begin{array}{cc} \sigma_{\alpha \beta } & 0\\ 0 & \sigma_{\alpha \beta } \end{array}\right)+\left(\begin{array}{cc} 0 & \tau_{xy}^{s}\\ \tau_{xy}^{s} & 0 \end{array}\right)=\sigma_{\alpha \beta }I_{s}+\tau_{\mathrm{dev}}^{s}$$

In this case, thermodynamics and mechanics are decoupled, since the former is contained in the diagonal elements of **σ** ^*s*^ whereas the latter only appears in the non-diagonal terms, making it possible to measure the material functions related to simple shear deformations in a direct and decoupled manner. As long as we are able to measure $\sigma_{xx}^{s}$ and $\sigma_{xy}^{s}$ independently, which is possible in practice (see Section 4.2.1), we can determine the state variable and the rheological material functions of the interface unequivocally, with fully decoupled raw data. When exploring complex interfaces, only under these very specific conditions (isothermal, planar interface, spatially homogeneous σ_*αβ*_), our experimental raw data can be directly interpreted as a measurement of the state variable or the rheological shear stress. When related to the applied flow kinematics, the latter yields the interface’s material functions.^14,79,80^

***Postulate***: In (linear) simple shear flows, thermodynamic and rheological effects are decoupled, and the shear material functions can be determined independently from σ_*αβ*_.

##### 3.5.2.2 Pure Shear

Pure shear deformations—also known as planar stretch or pure extension—are irrotational, area-conserving deformations with stretches of λ and 1/λ along the principal axes (*x* and *y* in Figure 8).^8,35,53,77,81^ Although not as common as simple shear, pure shear deformations are applied by the Quadrotrough interfacial rheometer^35,53^ for measuring interfacial shear material functions (see Tein et al.^35^ and the Supporting Information).

#### 3.5.3 Dilatational Rheology

A spatially homogeneous dilatation requires that the shape of each interface element remain constant while its area changes isotropically.^12,13,35^ The rheological dilatational stress should **not** to be confused with the Gibbs elasticity (see Section 3.4.2): when complex interfaces are expanded/compressed, both a thermodynamic response, due to the interface compressibility and manifested through changes in σ_*αβ*_ (i.e., Gibbs elasticity), and a rheological response, caused by the surface mechanically resisting the imposed area changes and quantified by $\tau_{\mathrm{iso}}^{s}$ , may arise. This difference can be more easily visualized by traveling to Flatland. Imagine that Flatlanders are holding their neighbors’ spring-arms, forming a network, and that the surface area of Flatland is changed. Whereas thermodynamic stresses (i.e., interfacial tension) are caused by changes in the population density, isotropic extra stresses (i.e., dilatational rheology) arise when the springs are extended or compressed past equilibrium. The two phenomena are inherently related, both physically (a change in the interface density leads to a change in the length of the springs) and mathematically (σ_*αβ*_ ***I***_s_ and $\tau_{\mathrm{iso}}^{s}$ are diagonal tensors), which explains why it is so difficult to experimentally separate thermodynamic and isotropic mechanical stresses. Despite these difficulties, interfacial dilatational rheology plays a crucial role in many biologically and industrially relevant applications.^12,82,83^

##### 3.5.3.1 Pure Dilatation

Consider a planar, isothermal, and insoluble interface, as depicted at the right of Figure 8, whose total area changes by a factor of *A*/*A*_0_.^8,13^ Since a pure dilatation is isotropic (i.e., $\tau_{xx}^{s}$ = $\tau_{yy}^{s}$ = $\tau_{\mathrm{iso}}^{s}$ ), $\tau_{\mathrm{iso}}^{s}$ and **σ** ^s^ can be expressed as scalars:

14 $$\sigma^{s}=\left(\begin{array}{cc} \sigma_{xx}^{s} & \sigma_{xy}^{s}\\ \sigma_{yx}^{s} & \sigma_{yy}^{s} \end{array}\right)=\left(\begin{array}{cc} \sigma_{\alpha \beta } & 0\\ 0 & \sigma_{\alpha \beta } \end{array}\right)+\left(\begin{array}{cc} \tau_{\mathrm{iso}}^{s} & 0\\ 0 & \tau_{\mathrm{iso}}^{s} \end{array}\right)=\left(\sigma_{\alpha \beta }+\tau_{\mathrm{iso}}^{s}\right)I_{s}=\sigma_{\mathrm{iso}}^{s}I_{s}$$

Even in these near-optimal experimental conditions, the thermodynamic and mechanical contributions are inevitably coupled and our measurement of $\sigma_{\mathrm{iso}}^{s}$ is a combination of both.

Knowing σ_*αβ*_ as a function of *A*/*A*_0_ allows us to determine $\tau_{\mathrm{iso}}^{s}$ from the experimentally measured $\sigma_{\mathrm{iso}}^{s}$ . To separate thermodynamic and rheological contributions in insoluble interfaces or soluble interfaces with Da ≫ 1, we can do the following:

(i) Obtain the true isotherm, following the precautions in Sections 3.4.1 and 4.1. With the thermodynamics of the interface fully determined from σ_*αβ*_(Γ)_*T*_, conduct the rheological experiment by applying the desired deformation and measuring $\sigma_{\mathrm{iso}}^{s}$ . For insoluble interfaces, Γ = Γ_0_ *A*_0_/*A* (subscripts indicate initial values for an un-deformed interface) and the rheological stress at different areas is calculated as $\tau_{\mathrm{iso}}^{s}\left(A\right)$ = $\sigma_{\mathrm{iso}}^{s}\left(\Gamma \left(A\right)\right)$ – $\sigma_{\alpha \beta }\left(\Gamma \left(A\right)\right)$ .^13,14,53,80^

(ii) Following the clever approach of Verwijlen et al.,^8^ perform the dilatational experiments at varying initial concentrations, Γ_0_, where the changes in $\sigma_{\mathrm{iso}}^{s}$ as a function of Γ are used to separate the thermodynamic and rheological contributions.^8,12^ Note that both approaches assume that the dilatational strain is spatially homogeneous, such that the local area change of each interface element is equal to *A*/*A*_0_.

***Postulate***: A pure dilatational deformation is isotropic and **σ** ^s^ can be expressed as a scalar, $\sigma_{\mathrm{iso}}^{s}$ , which is a combination of σ_*αβ*_ (thermodynamics) and $\tau_{\mathrm{iso}}^{s}$ (rheology).

When exploring the dilatational response of complex interfaces, only under these very specific conditions (homogeneous σ_*αβ*_, known isotherm, no Marangoni stress, kinematically pure dilatation, Bq ≫ 1, Da ≫ 1, constant *T*) is it straightforward to distinguish $\tau_{\mathrm{iso}}^{s}$ and σ_*αβ*_ from the measured stress, $\sigma_{\mathrm{iso}}^{s}$ . Remember that, if these effects cannot be fully decoupled and the true rheological response cannot be obtained, we must report the results of our interfacial rheology experiments in terms of **effective** material functions, as discussed in Section 3.1.

##### 3.5.3.2 Mixed Dilatational Deformations

Pure dilatations are challenging to achieve in practice, and only a few instruments can impose them reliably.^13,35,84,85^ Commercial devices, such as Langmuir–Pockels troughs and drop-based setups (see Section 4.3.1), are widely available and commonly used to probe dilatational responses. However, these instruments impose mixed deformations that combine shear and dilatation. For example, in the rectangular Langmuir–Pockels geometry, with an adequate choice of the reference frame, **σ** ^s^ is diagonal and given by

15 $$\sigma^{s}=\left(\begin{array}{cc} \sigma_{xx}^{s} & \sigma_{xy}^{s}\\ \sigma_{yx}^{s} & \sigma_{yy}^{s} \end{array}\right)=\left(\begin{array}{cc} \sigma_{\alpha \beta } & 0\\ 0 & \sigma_{\alpha \beta } \end{array}\right)+\left(\begin{array}{cc} \tau_{xx}^{s} & 0\\ 0 & \tau_{yy}^{s} \end{array}\right)=\sigma_{\alpha \beta }I_{s}+\tau_{\mathrm{iso}}^{s}+\tau_{\mathrm{dev}}^{s}$$

However, since the barriers’ motions induce a non-isotropic deformation, $\tau_{xx}^{s}$ and $\tau_{yy}^{s}$ are different and, therefore, deviatoric stresses arise. Thus, the deformation is not purely dilatational, but *A* ≠ *A*_0_ so it is not purely shear, either. In drop-based geometries, **σ** ^s^ can also be expressed as a diagonal tensor (along the drop’s principal axes), and a mixed deformation is similarly obtained.^61,86,87^

## 4 How Can You Measure?

We will now outline the equipment and experimental procedures required to properly measure the different contributions to the interfacial stress explained previously—interfacial tension and changes thereof using tensiometry, and material functions for shear and dilatational rheometry. We conclude with practical recommendations on how to obtain good data.

### 4.1 Tensiometry

#### 4.1.1 Instruments

In **force-based methods**, a measurement probe—usually a Wilhelmy plate/rod or a du Noüy ring—lies in contact with a (planar) interface, and a force transducer measures the vertical capillary force exerted by the interfacial stress on the probe contour. As a direct method, no data post-processing is required, and σ_*αβ*_ is calculated by dividing the measured force by the probe’s perimeter. A Petri dish or beaker can be used to hold the solution (see Figure 9a), ensuring that the probe is sufficiently far from the container bottom and side walls to avoid capillary effects. Alternatively, the sample can be placed in a Langmuir–Pockels trough. Specialized trough and plate designs are available for liquid–gas or liquid–liquid interfaces. Langmuir–Pockels troughs are also equipped with a pair of movable barriers positioned at opposite ends, which can be used to change the interface area, and thereby the concentration of insoluble species, by means of a stepper motor system (see Figure 9b).

**Figure 9:**
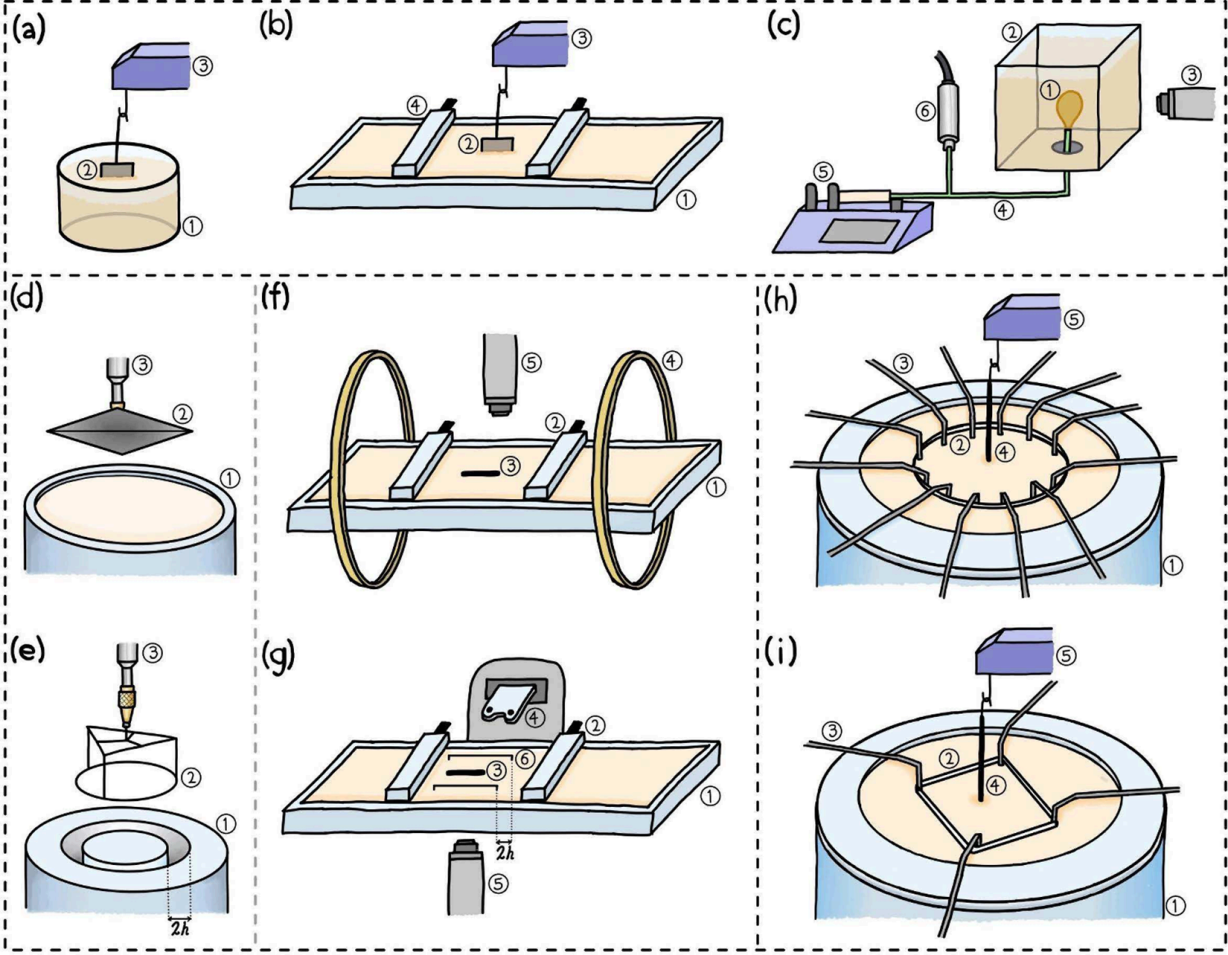
**Tensiometry:** (a,b) The interface is formed inside a container (a,1) or Langmuir–Pockels trough (b,1), and σ_*αβ*_ is measured using a Wilhelmy plate (2) and force transducer (3). A pair of movable barriers (b,4) compress the interface for isotherm measurements. (c) A drop/bubble (1) is formed inside a chamber (2), and a camera (3) records its profile. The drop is connected (4) to a pump (5) and a pressure transducer (6), used in CPT. **Shear rheometry:** (d, e) Rotational geometries. The interface is formed inside a measurement cell (1). A bicone (d,2) or DWR (e,2) probe is connected to the rheometer shaft (3). (f,g) ISRs. The interface is formed inside a Langmuir–Pockels trough (1) and Γ is controlled by a pair of movable barriers (2). A cylinder probe (3) translates along the interface, confined within a channel (g,6), driven by the magnetic force applied by a pair of Helmholtz coils (f,4) or permanent magnets (g,4). A camera (5) records the probe displacement. **Dilatational rheometry:** (h,i) The interface is formed inside a PTFE trough (1). An elastic barrier (2) surrounds a set of movable fingers (3) and the surface stress is measured by a Wilhelmy plate or rod (4) and force transducer (5).

In **drop/bubble-based methods**, a drop or bubble is formed at the tip of a capillary using a pump and immersed inside the desired solution (see Figure 9c). Depending on the density difference between the inner and outer bulk phases, either pendant or rising drops/bubbles are generated. Two methods are commonly employed: Axisymmetric Drop Shape Analysis (ADSA)^88–93^ and Capillary Pressure Tensiometry (CPT).^37,94–101^ Both require small sample volumes, which is an advantage over force-based methods for scarce or expensive samples, and rely on the Young–Laplace equation (refer to the Supporting Information for more information) to calculate σ_*αβ*_, assuming that (1) the bulk fluids are stationary, (2) there are no rheological stresses (i.e., **τ** ^s^ = 0), and (3) σ_*αβ*_ is spatially homogeneous.

In ADSA, the drop/bubble must be large enough to be deformed by gravity, making it possible to calculate σ_*αβ*_ directly from images of its contour.^61,89,102^ Shape-fitting techniques are widely available, either as part of commercial tensiometers or as open-source routines.^61,87,89^ CPT uses smaller (sub-hemispherical) geometries, where the drop/bubble contour is fit to a circle.^37,102–104^ Although image analysis is simplified, CPT is not commercially available and additionally relies on measurements of the drop/bubble pressure. ADSA and CPT are only valid when the interfacial stress is isotropic and can **only** be employed for simple interfaces or *unstrained* complex interfaces (i.e., where a rheological response has not yet been elicited).^6,61,86,87,105–109^ Drop-based methods can also be used to characterize complex interfaces, albeit with modifications discussed in Section 4.3.1 and in Danov et al.,^86^ Nagel et al.,^61^ Carvajal et al.,^105^ Jaensson and Vermant,^6^ and Rodríguez-Hakim et al.^87^

#### 4.1.2 Measurements

##### 4.1.2.1 Soluble (Gibbs) Interfaces

To understand the **adsorption/desorption kinetics** of soluble species, we must measure the time-dependent evolution of σ_*αβ*_. Sorption will stop once equilibrium is reached and σ_*αβ*_ reaches a constant value. The temporal evolution of σ_*αβ*_ (see Figure 10a) allows us to determine adsorption and desorption time scales, *t*_ads_ and *t*_des_, and calculate Da (see Section 3.3). Measurements are relatively easy to carry out with force-based methods, although drop-based methods are preferred for capturing initial transients and examining the influence of interfacial curvature on sorption time scales.^39,40^

**Figure 10:**
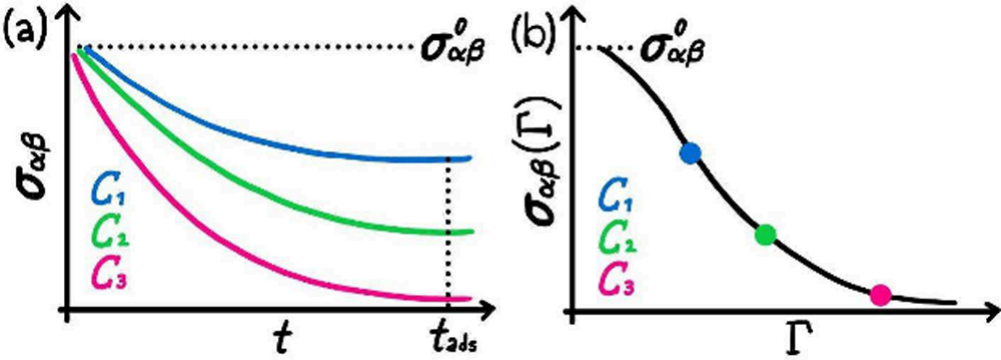
Tensiometry of soluble interfaces: (a) Adsorption kinetics are obtained from the temporal evolution of σ_*αβ*_ at different bulk concentrations, *C*_*i*_. (b) Adsorption isotherms are obtained from the equilibrium value of σ_*αβ*_ at varying *C*_*i*_, indicated by the colored points.

It is possible to create **adsorption isotherms** for soluble Flatlanders from the equilibrium points of adsorption measurements at different bulk concentrations, *C*_*i*_, as shown in Figure 10b, where kinetic models are required to obtain Γ from *C*_*i*_.^33,54,110^

##### 4.1.2.2 Insoluble (Langmuir) Interfaces

**Compression isotherms** can be obtained for insoluble interfaces, as outlined in Sections 2.1 and 3.4.1, where σ_*αβ*_ is measured as a function of Γ at constant *T*: σ_*αβ*_(Γ)_*T*_. Γ can be increased by either^111^ (1) spreading additional surface active material (see Section 4.4.2) to increase *N*_*s*_ at a fixed *A*, although this is not recommended because spreading on an already densely populated interface can induce out-of-equilibrium states with residual stresses, or (2) keeping *N*_*s*_ constant and quasi-statically compressing the interface to decrease *A*. We suggest spreading a dilute concentration of Flatlanders, such that Π_*αβ*_ < 1 mN/m, followed by a ∼1–10 min waiting period for sample equilibration, and a quasi-static compression at ∼1 mm^2^/min; however, this should be checked for different interfaces, e.g., by conducting compressions at different speeds, because, if the measured stress changes with barrier velocity, extra rheological stresses are also being measured. Langmuir–Pockels troughs with movable barriers are the gold standard for measuring compression isotherms. Using the minimum barrier speeds to ensure the slowest possible compression comes at the expense of having a very long measurement, where evaporation or contamination may compromise the data quality. Aside from continuous compressions, isotherms can also be obtained by applying small step compressions and waiting for the stress to relax to equilibrium.^13,14^ Upon relaxation, it is assumed that all viscoelastic stresses have dissipated completely (De ≪ 1), such that the measured stress corresponds exclusively to σ_*αβ*_. During a compression, Γ (in units of mol/cm^2^) can be calculated as a function of the applied area change, via Γ = Γ_0_ *A*_0_/*A*, where Γ_0_ is the initial spreading concentration at *A*_0_. If the Flatlanders’ molecular weight is known, a mean molecular area (MMA, in units of cm^2^/molec.) can also be calculated. If Γ_0_ is not known, surface coverage can be obtained from the initially spread mass (in units of g/cm^2^), or as a function of *A*/*A*_0_. Alternatively, for colloidal particles that can be visualized under a microscope, the surface coverage as a percentage of area occupied can be assessed via image analysis.

How can we be certain that we obtain a true isotherm and that the measured stress is purely thermodynamic (i.e., **σ** ^s^ ∼ σ_*αβ*_ ***I***_s_)? One way to check is by conducting isotherm experiments with two Wilhelmy plates oriented parallel and perpendicular to the movable barriers, which measure the *x* and *y* components of **σ** ^s^ (σ_*xx*_^s^ and $\sigma_{yy}^{s}$ , respectively, see Figure 11a).^13,112,113^ In principle, $\sigma_{xx}^{s}$ and $\sigma_{yy}^{s}$ can contain thermodynamic and mechanical stresses (i.e., $\sigma_{xx}^{s}$ = $\sigma_{\alpha \beta }$ + $\tau_{xx}^{s}$ and $\sigma_{yy}^{s}$ = $\sigma_{\alpha \beta }$ + $\tau_{yy}^{s}$ ). Since σ_*αβ*_ is a scalar, its value does not depend on the plate orientation (see Section 2.1);^112,114,115^ however, **τ** ^s^ is direction-dependent and, for mixed deformations such as those in a Langmuir–Pockels trough, $\tau_{xx}^{s}$ ≠ $\tau_{yy}^{s}$ (see Section 4.3.1). Thus, if $\sigma_{xx}^{s}$ = $\sigma_{yy}^{s}$ (as shown in Figure 11b and Figure 11c for Γ < Γ*), then $\tau_{xx}^{s}$ = $\tau_{yy}^{s}$ = 0; the measured stress is purely thermodynamic and a true isotherm is obtained. However, if there are clear and reproducible differences between compression curves ( $\sigma_{xx}^{s}$ ≠ $\sigma_{yy}^{s}$ , see Figure 11c for Γ > Γ*), surface rheological stresses are present and a true isotherm is **not** obtained.^13,80^

**Figure 11:**
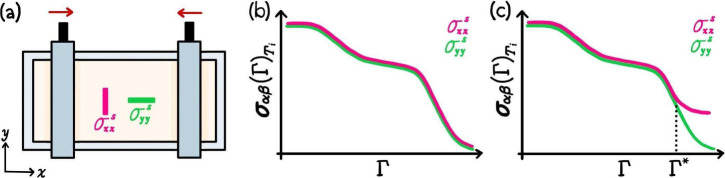
(a) A pair of Wilhelmy plates are placed parallel and perpendicular to the barriers in a Langmuir–Pockels trough. If $\sigma_{xx}^{s}$ = $\sigma_{yy}^{s}$ , rheological effects are absent and a true isotherm is being measured. (b) A true isotherm. (c) A true isotherm is obtained for Γ ≤ Γ*.

### 4.2 Shear Rheometry

#### 4.2.1 Instruments

##### 4.2.1.1 Rotational Flows

The first class of interfacial shear rheometers is based on rotational geometries that apply simple shear flows, where a measurement probe is placed at a gas–liquid or liquid–liquid interface and subjected to an angular displacement, while the torque on the probe is measured. Early designs using torsion wires date back to the 1970s, such as the knife-edge, disk, and biconical disk viscometers.^45,116,117^ Nowadays, rotational geometries are readily available for use with commercial bulk rheometers, which renders interfacial shear rheology increasingly popular. These configurations operate in the same manner as those used for bulk rheometry: stresses and strains are calculated from the torque and angular velocity of the probe, and material functions are defined for different kinematics. One of the first commercially available interfacial rotational geometries was the bicone,^118^ which operates as a 2D analogue of a concentric cylinder (Couette) geometry, shown in Figure 9d. Its main drawback is that it is relatively heavy, leading to a high geometry inertia, and has a large contact area with the bulk phases, making it suitable only for very strong interfacial layers.

To address these limitations, the double wall-ring (DWR)^75,119^ was developed as a 2D analogue of the double gap concentric cylinder, shown in Figure 9e. The DWR has a significantly smaller contact area with the subphases, greatly expanding the instrument precision. The DWR is placed inside an annular measurement cell, where the gap between the probe and cell walls is small (≲1 cm) to try to eliminate surface gradients in $\tau_{\mathrm{dev}}^{s}$ , which simplifies data analysis (see Section 4.2.3), while remaining larger than the capillary length to avoid curvature effects.^119^ Several modifications to the DWR have emerged to enable more specialized measurements, including small openings in the ring and measurement cell to allow their use on Langmuir–Blodgett troughs for measurements at controlled σ_*αβ*_.^120^ Such adaptations have enabled studies of microgels,^121^ glassy polymers,^13,122^ solid particles,^80^ and antibodies.^123^ DWRs constructed with alternative materials to improve heat transfer^124^ and adaptations to the measurement cell that allow for real-time visualization of the interface dynamics^125,126^ have been developed. Recently, the DWR has been coupled with neutron reflectometry for simultaneous structural–rheological characterization of interfacial layers.^127,128^

##### 4.2.1.2 Channel Flows

In the Interfacial Shear Rheometer (ISR), the rotational measurement probe is replaced by a thin magnetic rod that floats on the interface. An external magnetic field applies a force on the rod to create a simple shear flow, and its motion is tracked by a camera to quantify the deformation (see Figure 9f,g). The probe is confined longitudinally within a glass channel to impose a constant $\tau_{xy}^{s}$ , which simplifies data analysis (see Section 4.2.3).

In the ISR’s original design, the magnetic field was applied by a pair of Helmoltz coils^48,64,129^ (Figure 9f). Two aspects were improved in its most recent commercial version, the magnetic trap ISR^31,130^ (Figure 9g). First, the coils were replaced by a pair of permanent magnets, which enhance the instrument sensitivity by eliminating the centering force required in the coil-based design to keep the rod in place and also ease the operator’s work during probe positioning.^31^ Second, magnetic microwires (diameters ∼20 μm) can be used as probes in lieu of the standard magnetic rods, thus increasing Bq_η_ by reducing the characteristic length scales^130^ (see Section 4.2.3). These improvements render the magnetic trap ISR capable of measuring interfacial viscosities down to 10^–7^ Pa·s·m^–1^.^31,62^

Aside from their sensitivity, the main advantage of ISRs is that the sample container is a Langmuir–Pockels trough, allowing for measurements at controlled values of Γ or σ_*αβ*_. Combining microstructural visualization studies is very practical in these setups, due to the large surface area, and has been done for particle-laden interfaces^131^ and phospholipid monolayers using fluorescence.^132^ ISRs’ main disadvantage is that they are limited to small or oscillatory deformations, unlike rotational geometries where steady shear is also possible.

##### 4.2.1.3 Pure Shear Flows

The custom Quadrotrough rheometer (Figure 9i) can apply either pure shear, pure dilatational, or mixed deformations.^35,53^ For more information, see Tein et al.^35^ and Ashkenazi et al.^53^

##### 4.2.1.4 Other Techniques for Shear Flows

Other techniques, which we will not discuss in detail, include (a) contactless interfacial rheometry,^133,134^ where a flow at the interface is created by shearing one of the liquid bulk phases and the response is tracked by tracer particles; (b) microbutton measurements,^135^ where a ∼10 μm circular magnetic probe rotates on the interface plane; and (c) passive/active microrheology,^136,137^ which measure the diffusion/motion of a probe at the interface.^62,138,139^

#### 4.2.2 Measurements

##### 4.2.2.1 Oscillatory Tests

Oscillatory interfacial shear rheometry quantifies the viscous vs elastic character of a sample using sinusoidal strain and stress fields.^4,66,140^ The relationship between the amplitude and phase of the probe’s displacement and the amplitude and phase of the instrument torque are used to calculate the interfacial shear strain $\left(\gamma_{xy}^{s}\right)$ and rheological stress $\left(\tau_{xy}^{s}\right)$ , which are conveniently expressed in complex notation (i.e., $\gamma_{xy}^{s}$ = $\Delta \gamma_{0}^{s}\;e^{i\omega t}$ and $\tau_{xy}^{s}$ = $\Delta \tau_{0}^{s}\;e^{i\left(\omega t+\delta \right)}$ ). The outputs of the experiment are the amplitude ratio $\left(\Delta \gamma_{0}^{s}/\Delta \tau_{0}^{s}\right)$ and the phase lag (δ) between $\gamma_{xy}^{s}$ and $\tau_{xy}^{s}$ .^31,38,47,48,64,66,68^ Considering the precautions described in Section 4.2.3, one can find the shear material functions represented either as a complex viscosity, $\eta_{s}^{*}=\eta_{s}^{\prime }-i\eta_{s}^{\prime \prime }$ , or as a complex modulus, $G_{s}^{*}=G_{s}^{\prime }+iG_{s}^{\prime \prime }$ , where $G_{s}^{\prime }$ and $G_{s}^{\prime \prime }$ are the elastic (storage) and viscous (loss) shear moduli, respectively. Note that $\eta_{s}^{*}$ and $G_{s}^{*}$ can be functions of ω, $\Delta \gamma_{0}^{s}$ , and/or *t*.

Small-amplitude oscillatory shear (SAOS) tests are conducted within the linear viscoelastic regime (LVR), at small strain amplitudes, where the material functions are independent of Δγ_0_^s^. Common types of experiments include (i) strain or stress amplitude sweeps, where the prescribed strain or stress amplitude, $\Delta \gamma_{0}^{s}$ , is progressively increased at a fixed frequency, ω, until the upper limit of the LVR and onset of nonlinear response are reached (see Figure 12a); (ii) frequency sweeps, where ω is varied at a fixed $\Delta \gamma_{0}^{s}$ within the LVR, to probe the interfacial response at different time scales (see Figure 12b); (iii) Π_*αβ*_ sweeps, where changes in the material properties at constant ω and $\Delta \gamma_{0}^{s}$ are studied as Π_*αβ*_ is varied; and (iv) time sweeps, where the time-dependent behavior is studied at fixed ω and $\Delta \gamma_{0}^{s}$ (see Figure 12c). For soluble interfaces, time sweeps can monitor the development of viscoelasticity during adsorption, as has been done for proteins^118^ and bacterial biofilms.^141^

**Figure 12:**
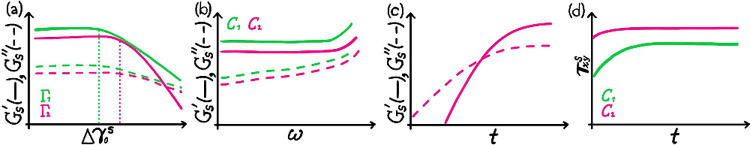
Examples of interfacial shear rheology experiments (analogous tests can be conducted for dilatation). (a) An oscillatory shear amplitude sweep tracks the evolution of $G_{s}^{\prime }$ and $G_{s}^{\prime \prime }$ as a function of $\Delta \gamma_{0}^{s}$ , the shear strain amplitude. Results can be obtained at varying Γ or *C* for insoluble and soluble interfaces, respectively. For dilatation, $\Delta \gamma_{0}^{s}$ is the area strain amplitude. (b) An oscillatory shear frequency sweep shows the evolution of $G_{s}^{\prime }$ and $G_{s}^{\prime \prime }$ , as a function of ω. Results are shown for a soluble interface at varying *C*. (c) An oscillatory shear time sweep reveals time-dependent rheological changes at the interface. (d) A step shear strain is applied at *t* = 0 and the temporal stress relaxation, $\tau_{xy}^{s}\left(t\right)$ , is monitored.

Sometimes, determining the rheological properties at larger strain amplitudes is desired.^142,143^ Data stemming from large-amplitude oscillatory shear (LAOS) experiments needs to be interpreted with caution: At large strains, $\tau_{xy}^{s}$ can exhibit non-sinusoidal responses and large variations in response to small changes in the operating parameters. In addition, normal stress differences and higher order harmonics may arise under these non-linear conditions, requiring constitutive models adapted for modest strain non-linearities.^144^

##### 4.2.2.2 Steady and Transient Tests

Akin to bulk rheology, there are many different material functions to interrogate the sample behavior beyond linearity. For instance, flow curves quantify the steady interfacial shear viscosity, $\eta_{s}\left({\mathrm{\gamma ̇}}_{xy}^{s}\right)$ , which is obtained from the ratio between the shear stress and shear rate, $\tau_{xy}^{s}/{\mathrm{\gamma ̇}}_{xy}^{s}$ . In start-up shear flows (i.e., application of a constant ${\mathrm{\gamma ̇}}_{xy}^{s}={\mathrm{\gamma ̇}}_{0}^{s}$ for *t* ≥ 0), a transient interfacial shear viscosity is defined as $\eta_{s}^{+}\left(t,{\mathrm{\gamma ̇}}_{0}^{s}\right)$ = $\tau_{xy}^{s}/{\mathrm{\gamma ̇}}_{0}^{s}$ . In step-strain tests (or stress–relaxation tests), a step shear displacement, $\gamma_{xy}^{s}=\gamma_{0}^{s}\delta \left(t\right)$ , is applied and a shear relaxation modulus, $G_{s}\left(t,\gamma_{0}^{s}\right)$ = $\tau_{xy}^{s}\left(t\right)/\gamma_{0}^{s}$ , is obtained upon cessation of movement, revealing the interface’s relaxation behavior (see Figure 12d). Alternatively, creep tests can be used to obtain the interface compliance, *J*_s_, where a constant $\tau_{xy}^{s}$ is applied for a given period of time, followed by cessation of the imposed stress, during which the material properties are measured.^52^ For more details, we refer to classical bulk rheology textbooks.^144–146^

#### 4.2.3 Data Analysis: Calculating Material Functions for Shear

As previously mentioned, the shear deformation mode allows one to decouple thermodynamics and mechanics, being this feature the great advantage of interfacial shear rheometric techniques. However, unless the interface is deformed very slowly, the interface motion will also generate a flow in the adjacent bulk phases, resulting in a coupling between the interfacial and bulk stresses, mathematically captured by eq 4. Imagine, for example, that we want to measure the shear properties of an interface formed on top of a very viscous subphase. The drag on the probe will be mainly governed by the high bulk phase viscosity, rather than by the resistance of the interface. Thus, it is important to calculate the relative importance of interfacial vs bulk stresses and determine whether bulk hydrodynamics can be safely neglected in our data analysis. There is not a magic formula to determine whether bulk momentum transport matters in our experiments *in advance*, but a scaling analysis can help us check that our data analysis assumptions are acceptable. To neglect bulk contributions, two conditions must be met:^47,70^ (1) bulk stresses should not influence $\tau_{\mathrm{dev}}^{s}$ , which, according to eq 4, means that $\tau_{\mathrm{dev}}^{s}$ should be spatially homogeneous (i.e., $\nabla_{s}\cdot \tau_{\mathrm{dev}}^{s}$ = 0), and (2) bulk drag should not affect the probe’s motion.

Separate shear Boussinesq numbers can be defined for conditions (1) and (2), using eq 11. To ascertain whether bulk hydrodynamics influences the interfacial flow field, we use the interfacial momentum balance (eq 4) to obtain scalings for τ^s^, σ^s^, and *l*,^70^ such that

16 $${\mathrm{Bq}}_{\eta }^{\left(1\right)}=\frac{\eta_{s}H}{\eta h^{2}}$$

where *H* is the depth of the bulk liquid phase and *h* is the distance between the probe (bicone, ring, or magnetic needle) and the shear channel wall, shown in Figure 9e,g. A derivation and discussion of eq 16 is provided in the Supporting Information and in Fitzgibbon et al.^70^ For oscillatory flows, $\eta_{s}^{*}$ can be used in lieu of η_s_. Only when ${\mathrm{Bq}}_{\eta }^{\left(1\right)}$ ≫ 1, bulk hydrodynamics does not influence the interfacial flow field, which has a simple analytical solution that depends on the probe’s velocity and *h*,^47,48,70,75^ facilitating the calculation of the material functions.

The raw data acquired by the bicone, DWR, and ISR rheometers is some measurement of the force applied on the probe (torque applied by the DWR rotor or magnetic field gradient in the ISR) and some measurement of the imposed deformation (angular displacement of the DWR or position of the needle in the ISR); therefore, the bulk and interfacial stresses must be obtained from the probe’s equation of motion, where a Boussinesq number is defined from the ratio of the interfacial to bulk forces on the probe:^31,47,48,63,64,68–70,72,74–76,130^

17 $${\mathrm{Bq}}_{\eta }^{\left(2\right)}=\frac{\eta_{s}}{\eta a}$$

where *a* is the probe radius. Details on the probe’s force balance equation and the derivation of eq 17 are provided in the Supporting Information. If ${\mathrm{Bq}}_{\eta }^{\left(2\right)}$ ≫ 1, interfacial forces are much more important than bulk forces, and bulk viscous drag will not influence the probe’s motion. If ${\mathrm{Bq}}_{\eta }^{\left(2\right)}$ ≈ 1, we must be extremely careful to use a physical model that accounts for the drag from the bulk.^31,47,48,63,72,74–76^ However, if ${\mathrm{Bq}}_{\eta }^{\left(2\right)}$ ≪ 1, the physical quantity we are trying to calculate (η_*s*_ or $\eta_{s}^{*}$ ) has very little influence on the probe’s motion, which means that the measurement will lie outside the rheometer’s operating range, as discussed in Tajuelo et al.,^31^ Renggli et al.,^63^ and Guzmán et al.^62^

Although ${\mathrm{Bq}}_{\eta }^{\left(2\right)}$ is the more common definition of Bq, keep in mind that both ${\mathrm{Bq}}_{\eta }^{\left(1\right)}$ and ${\mathrm{Bq}}_{\eta }^{\left(2\right)}$ must be large enough to neglect bulk effects. When either of these conditions is not met, numerical routines—known as Flow Field-Based Data Analysis (FFBDA) schemes—are needed to extract the bulk and interfacial hydrodynamics, using an iterative scheme to solve the Navier–Stokes equation, the interfacial momentum balance, and the probe’s equation of motion, along with a suitable constitutive model to relate $\tau_{\mathrm{dev}}^{s}$ to the interfacial strain and velocity.^31,47,48,63,68,72,74–76,130^ For linear models, the measured shear stress on the probe directly corresponds to the rheological stress, i.e., $\sigma_{xy}^{s}=\tau_{xy}^{s}$ (see Section 3.5.2).

Fortunately, software packages with analysis methods are available for practically all interfacial shear rheometers (bicone, DWR, and ISR) and for the most common types of measurements (oscillatory, etc.). Numerical routines for FFBDA, including the calculation of the flow profiles and the bulk phases correction, are available as open source,^47,72,74–76^ as summarized in Table 1. These routines are easy to use, and are designed so that the user can plug in the physical properties of their instrument (rotor inertia, probe’s mass, length, etc.) and upload the experimental raw data, and the material functions are calculated. The currently available FFBDA routines can run in a standard PC and solve for the hydrodynamic fields in a few seconds. Note that although some interfacial shear rheometers have their own data analysis routines, in general these only involve linear corrections and are therefore only adequate for interfaces with very high ${\mathrm{Bq}}_{\eta }^{\left(1\right)}$ and ${\mathrm{Bq}}_{\eta }^{\left(2\right)}$ . Let’s conclude this section by noting that the calculation of ${\mathrm{Bq}}_{\eta }^{\left(1\right)}$ and ${\mathrm{Bq}}_{\eta }^{\left(2\right)}$ requires knowledge of η_s_ or $\eta_{s}^{*}$ , which is a priori unknown. If we have a reasonable estimate for η_s_ or $\eta_{s}^{*}$ , then we can calculate ${\mathrm{Bq}}_{\eta }^{\left(1\right)}$ and ${\mathrm{Bq}}_{\eta }^{\left(2\right)}$ and gauge whether bulk hydrodynamics can be neglected. However, if no estimate is available, we must necessarily resort to FFBDA methods to calculate our interfacial material functions.

***Postulate***: Unless the Boussinesq numbers are very high (∼10^4^), we cannot rule out bulk hydrodynamics affecting our raw data, either due to a nonlinear velocity profile or to an increased drag on the probe. Since FFBDA routines are available and have very low computational cost, the best general practice is to use them in all cases.

**Table 1:** Interfacial Shear and Dilatational Rheometers and Data Analysis Schemes

Instrument	Deformation	Geometry	FFBDA?	References
Bicone	Simple shear	Planar	Yes, refs 47, 72, 74	^117^,^118^,^147^
DWR	Simple shear	Planar	Yes, refs 47, 75, 76	^75^,^148^
ISR	Simple shear	Planar	Yes, ref 47	^31^,^48^,^64^,^68^,^70^,^130^
Radial troughs	Dilatation	Planar	No	^13^,^14^,^32^,^84^,^85^
Quadrotrough	Dilatation, Pure shear	Planar	No	^35^,^53^
Langmuir trough	Mixed	Planar	No	^8,13,112,115,149^
Drop-based	Mixed	Curved	No	^37^,^61^,^86^,^87^,^150^

### 4.3 Dilatational Rheometry

Unlike for shear, where instruments and data analysis schemes are readily available to calculate the interfacial material functions, dilatational rheometers and their associated analysis routines are still under development, leading to many open areas of research.

#### 4.3.1 Instruments

##### 4.3.1.1 Isotropic Deformations of a Planar Interface

Custom dilatational rheometers that operate on planar interfaces with circular symmetry (i.e., radial troughs) have been developed based on earlier works.^151,152^ In the radial trough^13,32^ (see Figure 9h), the interface is created inside a PTFE container and enclosed by a flexible elastic band. A set of 12 radially arranged, movable “fingers” controls the interface area by changing the band’s perimeter. The area strain is calculated from the finger position, and the isotropic surface stress, $\sigma_{\mathrm{iso}}^{s}$ , is measured with a Wilhelmy rod.^12–14,52,80^ The radial trough has been modified to allow subphase exchange,^52^ enabling variations in the composition or pH of the bulk. An optical setup has also been integrated to enable microstructure visualization in particle-laden interfaces.^80^ A 3D-printable, miniaturized version was developed to interrogate small-scale structural evolution under isotropic compression via microscopy, where the area is controlled by a diaphragm mechanism.^85^ A similar miniaturized version, the Interfacial Dilatational Rheometer, relies on the pneumatic motion of the outer walls, and can be placed directly on a Langmuir–Pockels trough.^84^

An instrument similar to the radial trough is the Quadrotrough, with consists of a simplified design with four fingers, such that the surface area is a square^35,53^ (see Figure 9i). The advantage of the Quadrotrough is that it can apply both (pure) shear and dilatational strains, either independently or simultaneously, broadening the range of accessible deformations^35,53^ (recall that, as a general rule of thumb, it is more complicated to deconvolute transport, thermodynamic, and rheological effects and obtain true material functions under mixed flows). A Wilhelmy rod measures $\sigma_{\mathrm{iso}}^{s}$ under dilatation, and a pair of Wilhelmy plates aligned along the principal axes (*x* and *y* in Figure 8) measure $\sigma_{xx}^{s}$ and $\sigma_{yy}^{s}$ under shear.

Isotherms can be obtained on the radial troughs and Quadrotrough, and should yield the same results as other instruments since σ_*αβ*_ is a state variable^13^ (see Section 2.1).

##### 4.3.1.2 Mixed Area-Changing Flows

Often, techniques that were originally developed for interfacial tension measurements are inadvertently used to measure the rheological properties of complex interfaces. Many researchers use setups such as Langmuir–Pockels troughs and drop-based configurations described in Section 4.1.1 for conducting dilatational rheology measurements, as these instruments can impose area changes, are commercially available, and are (seemingly) user-friendly. However, this might not be appropriate for a few reasons.^8,61,87,112,114,149,153–155^

First, the imposed area deformations are often far from being isotropic in such devices (see Section 3.5.3). Langmuir–Pockels troughs apply uniaxial deformations where the area and shape of the interface change simultaneously, imposing both shear and dilatational strains.^8,112,114,154^ In addition, a shear stress gradient may develop in the direction parallel to the barrier axis, due to no-slip at the trough walls (although perfect slip is typically assumed), which leads to inhomogeneous strains that cannot be properly accounted for.^149^ For complex interfaces, only in the case of perfect wall slip and linear strains can the shear and dilatational contributions be separated, as explained in Petkov et al.^112^

Drop-based methods that use spherical geometries, such as CPT (see Section 4.1.1), have been claimed to impose kinematically pure dilatations. However, even if the drop/bubble remains spherical, it does not deform isotropically, since (i) shear deformations are present near the capillary tip,^61,86,87,107,150,153^ so a pure dilatation is never achieved, and (ii) the strain is spatially inhomogeneous, so techniques that assume a constant stress such as ADSA and CPT cannot be used for complex interfaces^61,87,156^ (see Section 4.1.1). Several methods, such as Capillary Meniscus Dynamometry (CMD),^61,86^ Pendant Capsule Elastometry (PCE),^109,156,157^ and others,^105,158^ have been developed to properly account for the spatial and directional dependence of **σ** ^s^, although these techniques cannot distinguish between the contributions from σ_*αβ*_ and **τ** ^s^. These methods can be complemented with techniques such as Stress Fitting Elastometry (SFE),^6,61,87^ which predict an *effective* *K*_s_ and *G*_s_ for elastic interfaces using **σ** ^s^ as an input. A promising avenue of research involves microbubbles driven by external triggers, where isotropic area changes are possible. This has been recently explored using ultrasound fields, which can act as ultra-high-frequency interfacial dilatational setups.^159^

Whenever possible, techniques that apply mixed flows should be avoided when studying interfacial rheological properties, and kinematically pure dilatation or shear should be applied instead. However, when the use of instruments that generate mixed flows is unavoidable—due to experimental convenience, equipment availability, or other constraints—it is crucial to understand which effects contribute to the measured stress response and whether results need to be reported in terms of *effective* material functions, as discussed in Section 3.1.

#### 4.3.2 Measurements

##### 4.3.2.1 Oscillatory Tests

As in shear (Section 4.2.2), oscillatory dilatational rheometry uses sinusoidal strain and stress signals to obtain the dilatational elastic and viscous moduli, $K_{s}^{\prime }$ and $K_{s}^{\prime \prime }$ (or, alternatively, $\kappa_{s}^{\prime \prime }$ and $\kappa_{s}^{\prime }$ ). Experiments analogous to SAOS can be conducted for small-amplitude oscillatory dilatation (SAOD), where care must be taken to impose a sinusoidal oscillation in the *area* strain, and not in the barrier position (for trough-based methods) or drop/bubble volume (for drop-based methods). Applications of interest may also lie beyond the LVR, such as mimicking oscillatory breathing cycles to characterize lung surfactant interfaces.^160,161^ The Hencky or neo-Hookean constitutive models can accommodate modest strain non-linearities, effectively extending the linear regime to larger area strains.^3,13,32,44^ As with LAOS, large-amplitude oscillatory dilatation (LAOD) experiments are used to obtain responses beyond linearity.^162,163^

##### 4.3.2.2 Transient Dilatation

As in shear, step compressions/expansions are used to extract a relaxation dilatational modulus, *K*_s_(*t*, Δ*A*/*A*_0_), and relaxation time(s).^52,164^ Interfacial dilatational rheometers are usually strain-controlled; for stress-controlled experiments (such as creep compliance tests), proportional–integral–derivative (PID) feedback controls are available in some commercial mixed-flow instruments and can also be implemented in custom devices to make the stress follow any prescribed function of time.^104^

#### 4.3.3 Data Analysis: Calculating Material Functions for Dilatation

Dilatational material functions can be calculated by applying appropriate constitutive models to relate the strain and rheological stress ( $\tau_{\mathrm{iso}}^{s}$ for pure dilatation and $\tau_{\mathrm{iso}}^{s}$ and $\tau_{\mathrm{dev}}^{s}$ for mixed flows, see Section 3.1). Recall that to extract $\tau_{\mathrm{iso}}^{s}$ from the measured stress, σ_*αβ*_ must be independently obtained—typically via an isotherm (see Sections 3.4.1 and 4.1.2)—and separated from **σ** ^s^. By analogy to shear, a dilatational Boussinesq number is defined from eq 11,

18 $${\mathrm{Bq}}_{\kappa }=\frac{\kappa_{s}H}{\eta R^{2}}\gg 1$$

where *H* is defined as in eq 16 and *R* is the trough radius or side length. If Bq_κ_ ≫ 1, a uniform dilatational strain is obtained;^32^ however, if Bq_κ_ is not ≫ 1, not only will bulk hydrodynamics influence the interfacial flow but also shear contributions may arise due to an inhomogeneous interfacial strain field.^32,165^ FFBDA routines (see Section 4.2.3) have not yet been implemented in dilatational rheometry; thus, Bq_κ_ ≫ 1 is required by both trough- and drop-based setups (in this case, *R* is equal to the drop radius). For the radial troughs and Quadrotroughs, this poses an important constraint on the types of interfaces that can be measured, as it requires κ_s_ (or $\kappa_{s}^{*}$ ) ≳ 10^–2^ Pa·s·m.

In the radial troughs and Quadrotroughs the deformation is applied by a band around the interface perimeter and the stress is measured by a probe near the center of the geometry. Strong interfacial layers and slow deformations are necessary so that the strain imposed by the band can propagate instantaneously to the trough’s center. Moreover, the interface must be viscoelastic enough to eliminate surface damping effects, requiring Bq, De ≫ 1.^166,167^ Slow deformations (frequencies ≲ 0.1 Hz)^84^ are also necessary to reduce Marangoni flows^32^ and mitigate gravity–capillary waves,^84^ which introduce errors in the measured $\sigma_{\mathrm{iso}}^{s}$ .

### 4.4 General Experimental Recommendations

#### 4.4.1 Before Starting Your Experiments

Fluid–fluid interfaces are highly prone to contamination, and even tiny amounts of a contaminant that could go unnoticed in bulk experiments can be disruptive for the measurement of interfacial properties.^168^ It is paramount to ensure the cleanliness of the equipment, such as troughs, probes, and geometries that are in direct contact with the sample. This is achieved through repetitive cycles of washing and rinsing with an appropriate solvent and copious amounts of ultra-purified water.^80,169^ Flaming the Wilhelmy probe after the washing and rinsing cycles helps to burn off any organic impurities that may have remained on the surface. Contamination can also come from the glassware and utensils used to prepare samples, so we strongly recommend keeping a set of bottles, vials, and glass syringes for a specific use.

Working with high purity grade chemicals is important (>99.5%), especially for preparing samples. We recommend purifying organic compounds such as alkanes by flowing them through an aluminum oxide column. For insoluble species such as particles, cleaning the source suspension prior to spreading can improve the quality of the measured data. Cleaning protocols include repetitive cycles of centrifugation, supernatant removal, addition of clean continuous phase, and redispersion. This removes contaminants that could be present in commercial samples or samples that have been stored for long.^170^

One way to check the cleanliness of a Langmuir interface is by carrying out a “clean” compression with a pristine, uninhabited interface before spreading the surface active compound. For a compression on a clean interface, Π_*αβ*_ ≈ 0 mN/m; if deviations of ∼0.2 mN/m occur, impurities are likely present. Sometimes, aspiration under vacuum can remove the impurities. For certain interfaces, especially those exhibiting phase transitions, measured isotherms can be compared to other published works (e.g., hexadecanol^63,171,172^ ) as a validation step. For compounds that are large enough to be visualized under a microscope, contamination can sometimes be evident, as surfaces will exhibit an inhomogeneous structure and coverage.^168^ When working with a new type of Flatland Inhabitant, or in experiments where direct visualization is not possible, assessing interface cleanliness becomes more challenging. We recommend repeating each experiment at least three times to ensure reproducibility.

As interfacial scientists, we often deal with extremely small stresses and deformations; thus, accurate equipment calibrations are essential, especially when operating near the instruments’ sensitivity limits.^63^ Standard calibration procedures in commercial rotational shear rheometers include air bearing checks, torque calibrations, rotational mappings, and oscillatory mappings. All components should be properly aligned and leveled (e.g., trough, Wilhelmy balance, rheometer geometry). Instruments should ideally be placed on anti-vibration tables and enclosed inside cabins to mitigate external disturbances, minimize air drafts, reduce evaporation, and protect against particulates.

Having good temperature control is paramount for accurately measuring interfacial properties. Trough-based instruments usually contain connections to recirculating water baths, whereas rotational shear rheometers include embedded Peltier plate systems. A thermocouple can be used to measure the interfacial temperature; however, due to its finite width, it also measures the temperature of the adjacent bulk phases, often leading to an overestimation due to evaporative cooling. A more reliable approach is to use an infrared pyrometer, which measures the interface’s thermal radiation and thus provides a more accurate determination of its temperature.^79^ Thermalization of the interface above room temperature will accelerate evaporation and can cause condensation to appear on the instrument, especially when the interface is large in area, which might affect the optics and hinder visualization.

#### 4.4.2 The Art of Interface Preparation

To prepare a Langmuir interface, a dilute concentration of Flatlanders is dispersed in a volatile compound and the solution is added dropwise to the interface using a 20–100 μL syringe. Droplets of solution are formed at the syringe tip, close to the interface, and carefully released to ensure that they spread on the interface rather than sink into the bulk. This is repeated several times across the whole interfacial area, until the total spreading volume is deposited. This procedure is highly sensitive, as the accuracy of the spreading directly affects the reproducibility and quality of the results across experiments, so it is important to establish a consistent and reliable spreading protocol. The solvent will rapidly evaporate, leaving a known amount of Flatlanders irreversibly adsorbed at the interface, where Marangoni flows assist in homogeneously distributing them across the available area.

A well controlled spreading protocol minimizes material loss to the subphase and guarantees that the intended initial surface concentration, Γ_0_, is achieved. The volatile solvent is chosen based on its compatibility with the Flatlanders and immiscibility with the bulk phases. The most common solvents are chloroform, toluene, isopropanol, and other alcohols (ethanol, methanol). Samples in which Flatlanders are molecules (e.g., fatty acids, lipids), must be completely soluble in the spreading solvent, at bulk concentrations between 10^–2^ and 10 mg/mL. For solid particles, a primary dispersion is prepared at a known concentration (typically as an aqueous suspension), and then mixed with a volatile compound to sufficiently lower the interfacial tension but *not* dissolve the material (e.g., polystyrene particles would be completely dissolved by chloroform, so a solvent like isopropanol should be used instead). A 1:1 suspension:solvent ratio is typically used.

The value of Γ_0_ depends on the ranges of Γ one wants to achieve during an experiment. However, Γ_0_ should be chosen to be as low as possible, to avoid aggregation at the interface and the formation of metastable structures.^18^ A few adaptations to the spreading protocol have enabled the creation of very dense crystalline particle layers.^173,174^ This is possible for μm-sized particles that have a high binding energy to the surface, but might be difficult to reproduce for other species. Thus, the most convenient way to achieve a high coverage is by spreading a low interfacial concentration, followed by a slow, quasi-static compression to the desired Γ_0_. Shear rheometers containing Langmuir–Pockels setups, such as the ISR and adapted DWR geometries, have enabled the study of the shear properties as a function of Γ for different interfaces, such as those with fatty acids,^79^ lipids,^120^ glassy polymers,^13,175^ monoclonal antibodies,^123^ spherical particles,^80^ and microgels.^121^

Subphase exchange protocols are very useful to create interfaces under specific conditions, such as the assembly of insoluble layers^52,176^ or the activation of stimuli-responsive systems.^148,177,178^ Commercial geometries are available for rotational shear and pendant drop rheometers, but most subphase exchange configurations are custom-made. Care must be taken to maintain low flow rates that minimize disturbances to the interface and ensure that enough total volume is exchanged, so that the initial bulk phase is completely replaced.

The preparation of soluble interfaces is straightforward, since all we must do is wait for the compounds to diffuse through the bulk and adsorb at the interface. For soluble interfaces, measurements should begin as quickly as possible to capture the adsorption dynamics. For techniques involving flat interfaces, a delay of ∼20 s is expected between the time the interface is formed inside the sample container, the probe is positioned, and the measurement is started. For drop-based methods, this delay can be reduced to ∼5 s.

#### 4.4.3 Setting Up Your Experiment

Measurement probes are very delicate pieces of equipment, so be extra careful when handling them and always check that the geometries are not bent or broken. A crucial aspect of experiments is the correct placement of the measurement probe at the interface. For measurements involving Wilhelmy plates or rods, a near-zero contact angle at the probe surface must be ensured for correct force measurements. Visually inspect that the interface properly wets the probe along its entire perimeter and tare the force transducer prior to lowering the probe toward the interface. A fine balance must be struck between dipping the plate/rod too deeply inside the bulk phase, since buoyancy will affect the force balance reading, and not dipping it enough, since evaporation may lead to dewetting. For liquid–liquid interfaces, make sure that the top of the plate is fully submerged within the top phase to avoid contributions from the top phase–air interfacial tension. Plates of smaller heights are very practical for liquid–liquid interfaces and are commercially available.

The correct “pinning” of the interface to the sample containers and measurement probes is crucial to guarantee accurate kinematic conditions. For instance, the DWR geometry has a diamond-shaped cross section to ensure that the contact angle between the interface and ring is ∼90°. Troughs are usually made of PTFE to prevent capillary effects, since these can lead to the measurement of an apparent elasticity even for clean interfaces.^63^ In drop-based methods, pinning of the interface to the capillary tip ensures a no-slip condition. In experiments involving Langmuir–Pockels troughs with movable barriers, leakage of the interface around the barriers must be avoided, which leads to an uncontrolled loss of Flatlanders. Leakage can be monitored by placing a second Wilhelmy balance on the outer (clean) interface. Ribbon troughs are a useful alternative for interfaces that present sustained leakage.

Always keep an eye on your experiments and monitor changes in the fluid level, pinning, leakage, or adhesion of materials to the measurement geometries. In general—and we cannot stress this enough—familiarize yourself with the instrument, its software, and the measurement protocols. For example, make sure that the dimensions input in all software (e.g., DWR radius, Wilhelmy plate perimeter) are correct.

#### 4.4.4 After Your Experiments

When an experiment is finished, clean the equipment following the protocol in Section 4.4.1 and carefully store the measurement probes. Always check your data after each measurement: Are the measured/applied torque and/or displacements within the instrument operating windows? Do the applied stresses or strains correspond to their prescribed values? Do FFBDA schemes need to be used for data analysis?

## 5 Conclusions

In this Tutorial, we presented a comprehensive Roadmap for experimentally navigating the complex landscape of Flatland. We discussed the intricate interplay between thermodynamics, transport, and rheological effects at fluid–fluid interfaces, outlining their physical origins and explaining their mathematical coupling through the interfacial stress balance equation (Section 2.5). Whereas the thermodynamic interfacial tension, σ_*αβ*_, is a state variable that does not depend on the flow and deformation history of the interface (Section 2.1), rheological contributions are path-dependent (Section 2.4). Any attempt to characterize the rheology of complex interfaces must carefully consider when and how thermodynamic and transport contributions can be successfully decoupled from the interfacial mechanics.

In Section 3.1 we introduced material functions as the physical quantities that summarize the rheological behavior of our interface, and how constitutive models are used to obtain them. Although the output of an interfacial rheology experiment is one or more components of the interfacial stress tensor, **σ** ^s^ (thermodynamics + mechanics), the calculation of true material functions requires the rheological component, **τ** ^s^ (mechanics). We explained the importance of isotherms for determining σ_*αβ*_, which can be subtracted from **σ** ^s^ to obtain **τ** ^s^. In Sections 3.4 and 4.1 we explained that isotherms must be measured under quasi-static conditions (De ≪ 1) and can be obtained for insoluble interfaces or soluble interfaces with Da ≫ 1. When thermodynamics and rheology cannot be decoupled, effective material functions should be reported instead (Section 3.1).

We discussed in Section 3.5 that kinematically pure and viscometric deformations should be applied in both shear (shape-changing, area-preserving) and dilatational (area-changing, shape-preserving) rheometry. For interfacial shear flows (Section 4.2), we learned that interfacial tension contributions can be decoupled from mechanical effects, but that bulk hydrodynamics must be taken into account when ${\mathrm{Bq}}_{\eta }^{\left(1\right)}$ or ${\mathrm{Bq}}_{\eta }^{\left(2\right)}$ is not very high (∼10^4^). In this case, FFBDA schemes must be used to obtain **τ** ^s^ from the rheometer’s raw data, where user-friendly routines for the most common commercial interfacial shear rheometers (bicone, DWR, and ISRs) are available as open-source. Custom instruments have been developed for dilatation (Section 4.3), although interfacial tension and mechanics cannot always be decoupled. Since FFBDA schemes are not yet available for dilatation, experiments are limited to Bq_κ_ ≫ 1. Although we strongly recommend techniques with well-defined dilatational kinematics, this is not always feasible due to the lack of commercially available instruments; thus, instruments that apply mixed flows, such as drop-based devices or Langmuir–Pockels troughs, may need to be used instead.

Finally, in Section 4.4, we shared a few practical insights for conducting interfacial rheology experiments, which we hope can assist the reader in improving data quality and avoiding time-consuming mistakes. In the end, Interfacial Rheometry comes down to a balance between what we want to know and what is feasible in practice. While this Tutorial tries to break down, quantify, and systematize this process as much as possible, the design of any interfacial rheology experiment, inevitably, relies on the judgment of the researcher.

## Supplementary Material



## Notes

Tables of nomenclature, additional information on pure shear deformations, additional details on the shear Boussinesq numbers, and further details on drop/bubble-based methods (PDF)
