# Supplementary material for: How to Successfully Navigate Flatland: A Tutorial on the Rheometry of Fluid–Fluid Interfaces
Source: Langmuir. 2026 Jul 14;42(29):20865–87. doi: 10.1021/acs.langmuir.6c02098 (PMC13421968; doi:10.1021/acs.langmuir.6c02098)
Supplement: Supplementary file 1 [file la6c02098_si_001.pdf]

Supplemental Information
How to successfully navigate flatland: A tutorial on the rheometry of fluid-fluid interfaces

Mariana Rodríguez-Hakim,[†] Alexandra Alicke,[‡] and Javier Tajuelo[¶]

[†]*Departamento de Física Fundamental, Universidad Nacional de Educación a Distancia (UNED), Las Rozas de Madrid 28232, Spain*

[‡]*Department of Mechanical Engineering, Eindhoven University of Technology, P.O. Box 513, 5600 MB Eindhoven, The Netherlands*

[¶]*Departamento de Física Interdisciplinar, Universidad Nacional de Educación a Distancia (UNED), Las Rozas de Madrid 28232, Spain*

E-mail:

1 Relevant tables of nomenclature

Table S1: Nomenclature for interfacial rheology

Symbol	Name	Definition	SI Units
∇_{s}	Surface gradient operator	–	1/m
$\boldsymbol{n}$	Surface unit normal vector	–	–
$\boldsymbol{I}_{\mathrm{s}}$	Surface (2-dimensional) identity tensor	–	–
T	Temperature	–	K
N_s	Number of species adsorbed at the interface	–	–
A	Interfacial area	–	m^2
Γ	Interfacial concentration	$\Gamma = N_s/A$	$1/\mathrm{m}^2$
$\sigma^0_{\alpha\beta}$	Interfacial tension of a clean interface	$\sigma^0_{\alpha\beta}(T)$	N/m
$\sigma_{\alpha\beta}$	Interfacial tension	$\sigma_{\alpha\beta}(T,\Gamma)$	N/m
$\Pi_{\alpha\beta}$	Surface pressure	$\Pi_{\alpha\beta}(T,\Gamma) = \sigma^0_{\alpha\beta}(T) - \sigma_{\alpha\beta}(T,\Gamma)$	N/m
K_{Π}	Gibbs elasticity	$K_{\Pi} = \partial\sigma_{\alpha\beta}/\partial \ln A$	N/m
$\boldsymbol{\sigma}^{\mathrm{s}}$	Interfacial stress tensor	$\boldsymbol{\sigma}^{\mathrm{s}} = \sigma_{\alpha\beta}(T,\Gamma)\,\boldsymbol{I}_{\mathrm{s}} + \boldsymbol{\tau}^{\mathrm{s}}$	N/m
$\boldsymbol{\tau}^{\mathrm{s}}$	Interfacial extra stress tensor	–	N/m
$\boldsymbol{\tau}^{\mathrm{s}}_{\mathrm{iso}}$	Isotropic (dilatational) component of the interfacial extra stress tensor	$\boldsymbol{\tau}^{\mathrm{s}}_{\mathrm{iso}} = \mathrm{tr}\left(\boldsymbol{\tau}^{\mathrm{s}}/2\right)\boldsymbol{I}_{\mathrm{s}}$	N/m
$\boldsymbol{\tau}^{\mathrm{s}}_{\mathrm{dev}}$	Deviatoric (shear) component of the interfacial extra stress tensor	$\boldsymbol{\tau}^{\mathrm{s}}_{\mathrm{dev}} = \boldsymbol{\tau}^{\mathrm{s}} - \boldsymbol{\tau}^{\mathrm{s}}_{\mathrm{iso}}$	N/m
$\boldsymbol{\gamma}^{\mathrm{s}}$	Interfacial strain tensor	–	–
$\dot{\boldsymbol{\gamma}}^{\mathrm{s}}$	Interfacial rate-of-strain tensor	–	1/s
$\boldsymbol{v}^{\mathrm{s}}$	Interfacial velocity vector	–	m/s
λ	Viscoelastic relaxation time	–	s
η_{s}	Interfacial shear viscosity	$\boldsymbol{\tau}^{\mathrm{s}}_{\mathrm{dev}} = f\left(\eta_{\mathrm{s}}, \dot{\boldsymbol{\gamma}}^{\mathrm{s}}\right)$	Pa m s
κ_{s}	Interfacial dilatational viscosity	$\boldsymbol{\tau}^{\mathrm{s}}_{\mathrm{iso}} = f\left(\kappa_{\mathrm{s}}, \dot{\boldsymbol{\gamma}}^{\mathrm{s}}\right)$	Pa m s
η^*_{s}	Complex interfacial shear viscosity	$\boldsymbol{\tau}^{\mathrm{s}}_{\mathrm{dev}} = f\left(\eta^*_{\mathrm{s}}, \boldsymbol{\gamma}^{\mathrm{s}}, \dot{\boldsymbol{\gamma}}^{\mathrm{s}}\right)$ $\eta^*_{\mathrm{s}} = \eta'_{\mathrm{s}} + i\eta''_{\mathrm{s}}$	Pa m s
κ^*_{s}	Complex interfacial dilatational viscosity	$\boldsymbol{\tau}^{\mathrm{s}}_{\mathrm{iso}} = f\left(\kappa^*_{\mathrm{s}}, \boldsymbol{\gamma}^{\mathrm{s}}, \dot{\boldsymbol{\gamma}}^{\mathrm{s}}\right)$ $\kappa^*_{\mathrm{s}} + \kappa'_{\mathrm{s}} + \kappa''_{\mathrm{s}}$	Pa m s
G_{s}	Interfacial shear modulus	$\boldsymbol{\tau}^{\mathrm{s}}_{\mathrm{dev}} = f\left(G_{\mathrm{s}}, \boldsymbol{\gamma}^{\mathrm{s}}\right)$	N/m
K_{s}	Interfacial dilatational modulus	$\boldsymbol{\tau}^{\mathrm{s}}_{\mathrm{iso}} = f\left(K_{\mathrm{s}}, \boldsymbol{\gamma}^{\mathrm{s}}, \dot{\boldsymbol{\gamma}}^{\mathrm{s}}\right)$	N/m

K_s^*	Complex interfacial dilatational modulus	$\boldsymbol{\tau}^{s}_{\text{iso}} = f\left(K_s^*, \boldsymbol{\gamma}^{s}\right)$<br>$K_s^* + K_s' + K_s''$	N/m
G_s^*	Complex interfacial shear modulus	$\boldsymbol{\tau}^{s}_{\text{dev}} = f\left(G_s^*, \boldsymbol{\gamma}^{s}, \dot{\boldsymbol{\gamma}}^{s}\right)$<br>$G_s^* = G_s' + G_s''$	N/m
η_s^{eff}	Effective interfacial shear viscosity	$\boldsymbol{\sigma}^{s} = f\left(\eta_s^{\text{eff}}, \dot{\boldsymbol{\gamma}}^{s}\right)$	Pa m s
κ_s^{eff}	Effective interfacial dilatational viscosity	$\boldsymbol{\sigma}^{s} = f\left(\kappa_s^{\text{eff}}, \dot{\boldsymbol{\gamma}}^{s}\right)$	Pa m s
G_s^{eff}	Effective interfacial shear modulus	$\boldsymbol{\sigma}^{s} = f\left(G_s^{\text{eff}}, \boldsymbol{\gamma}^{s}\right)$	N/m
K_s^{eff}	Effective interfacial dilatational modulus	$\boldsymbol{\sigma}^{s} = f\left(K_s^{\text{eff}}, \boldsymbol{\gamma}^{s}\right)$	N/m

Table S2: Relevant nomenclature for bulk rheology

**Symbol**	**Name**	**Definition**	**SI Units**
$\boldsymbol{\nabla}$	Gradient operator	–	1/m
$\boldsymbol{I}$	3-dimensional identity tensor	–	–
P	Pressure	–	Pa
$\boldsymbol{\sigma}$	Bulk stress tensor	$\boldsymbol{\sigma} = -P\boldsymbol{I} + \boldsymbol{\tau}$	N/m^2
$\boldsymbol{\sigma}^{(1)}$	Bulk stress tensor in fluid (1)	–	N/m^2
$\boldsymbol{\sigma}^{(2)}$	Bulk stress tensor in fluid (2)	–	N/m^2
$\boldsymbol{\tau}$	Bulk extra stress tensor	$\boldsymbol{\tau} = 2\eta\boldsymbol{D}$	N/m^2
$\boldsymbol{D}$	Bulk rate of strain tensor	$\boldsymbol{D} = \left(\boldsymbol{\nabla v} + (\boldsymbol{\nabla v})^T\right)/2$	N/m^2
$\boldsymbol{v}$	Bulk velocity vector	–	m/s
η	Bulk shear viscosity	$\boldsymbol{\tau} = 2\eta\boldsymbol{D}$	Pa s

Table S3: Relevant dimensionless groups

**Symbol**	**Name**	**Formula**	**Definition**
Da_a	Adsorption Damköhler number	$\text{Da}_\text{a} = t_{\text{ads}}/t_{\text{proc}}$	Adsorption vs. Process timescales
Da_d	Desorption Damköhler number	$\text{Da}_\text{d} = t_{\text{des}}/t_{\text{proc}}$	Desorption vs. Process timescales
De	Deborah number	$\text{De} = \lambda\,\dot{\gamma}$	Viscoelastic relaxation vs. deformation timescale
$\text{Bq}_\eta^{(1)}$	Shear Boussinesq number	$\text{Bq}_\eta = \eta_s/\left(\sqrt{\eta\,\rho\,\omega}\,h^2\right)$	Interfacial shear stress vs. Bulk shear stress
$\text{Bq}_\eta^{(1)}$	Shear Boussinesq number	$\text{Bq}_\eta = \eta_s/\left(a\,\eta\right)$	Interfacial drag force vs. Bulk drag force
Bq_κ	Dilatational Boussinesq number	$\text{Bq}_\kappa = \kappa_s/\left(a\,\eta\right)$	Interfacial dilatational stress vs. Bulk stress

2 Pure shear deformations

Under pure shear, the interface is elongated in one direction and compressed in the perpendicular direction, as shown in Fig. 9 of the Tutorial[1–4]. As mentioned in the Tutorial, the magnitudes of the elongation and compression must be selected such that the area of each interface element remains constant. Thus, if the ratio of the final vs. initial length along the extensional axis (the y axis in Fig. 9) is λ, then the ratio of the final vs. initial length along the compressional axis (the x axis in Fig. 9) is $1/\lambda$.

For an appropriate reference frame, as depicted in Fig. 9 of the Tutorial, where the coordinate axes are aligned with the principal axes of stretch, we obtain that

$$\boldsymbol{\tau}^{\mathrm{s}}_{\mathrm{dev}} = \begin{pmatrix} \tau^s_{xx} & 0 \\ 0 & \tau^s_{yy} \end{pmatrix}. \tag{1}$$

Unlike for simple shear, where a deviatoric deformation causes a change in shape, in pure shear a normal stress difference (i.e., $\tau^s_{xx} - \tau^s_{yy} \neq 0$) is what leads to the shape change. Considering the total stress tensor $\boldsymbol{\sigma}^s$, we obtain that

$$\boldsymbol{\sigma}^s = \begin{pmatrix} \sigma^s_{xx} & \sigma^s_{xy} \\ \sigma^s_{yx} & \sigma^s_{yy} \end{pmatrix} = \begin{pmatrix} \sigma_{\alpha\beta} & 0 \\ 0 & \sigma_{\alpha\beta} \end{pmatrix} + \begin{pmatrix} \tau^s_{xx} & 0 \\ 0 & \tau^s_{yy} \end{pmatrix} = \sigma_{\alpha\beta}\boldsymbol{I}_{\mathrm{s}} + \boldsymbol{\tau}^{\mathrm{s}}_{\mathrm{dev}}. \tag{2}$$

Moreover, pure shear flows are irrotational (unlike simple shear), which means that large shear strains can be applied to probe nonlinear behavior and measure the nonlinear extensional properties of fluid interfaces, as done in extensional rheometry[5,6]. In contrast, simple shear is a combination of i) area-conserving extension (elongation and compression) along the principal axes and ii) rotation, where only extension contributes to the surface stress[1,7–11].

Unlike simple shear where the surface tension and the rheological stress are independently measured, in pure shear the thermodynamic and the mechanical contributions are measured

simultaneously, since

$$\sigma^s_{xx} = \sigma_{\alpha\beta}(T, \Gamma) + \tau^s_{xx}, \tag{3}$$

$$\sigma^s_{yy} = \sigma_{\alpha\beta}(T, \Gamma) + \tau^s_{yy}. \tag{4}$$

Although this may seem like a problem at first glance, we can still determine the state variable and the interfacial material functions as long as $\sigma_{\alpha\beta}$ is spatially homogeneous. Remember, since neither Γ or T change, $\sigma_{\alpha\beta}$ remains constant and the measured *changes* in $\boldsymbol{\sigma}^s$ are exclusively a product of shear rheological effects[4].

3 Additional details on the shear Boussinesq numbers

3.1 The influence of bulk hydrodynamics on the interfacial shear stress

In all rotational and channel-based interfacial shear rheometers that apply simple shear deformations, the interfaces are assumed to be isothermal, planar, and spatially homogeneous. Therefore, Marangoni and capillary contributions are absent and the interfacial momentum balance equation (Eq. 5 in the Tutorial) reduces to a balance between the bulk and interfacial rheological stresses:

$$\left(\boldsymbol{\sigma}^1 - \boldsymbol{\sigma}^2\right) \cdot \boldsymbol{n} = \nabla_{\mathrm{s}} \cdot \boldsymbol{\tau}^{\mathrm{s}}_{\mathrm{dev}}, \tag{5}$$

where, using the linear Boussinesq-Scriven model, the only unique nonzero component of $\boldsymbol{\tau}^{\mathrm{s}}_{\mathrm{dev}}$ is τ^{s}_{xy} (recall that $\boldsymbol{\tau}^{\mathrm{s}}_{\mathrm{dev}}$ is a symmetric tensor, so $\tau^{\mathrm{s}}_{xy} = \tau^{\mathrm{s}}_{yx}$). If $\nabla_{\mathrm{s}} \cdot \boldsymbol{\tau}^{\mathrm{s}}_{\mathrm{dev}} = \mathbf{0}$, then $\boldsymbol{\tau}^{\mathrm{s}}_{\mathrm{dev}}$ has a simple analytical solution that depends on the coordinate system. For instance, for a flat interface oriented in the z-direction, the Boussinesq-Scriven constitutive model reveals that $\tau^{\mathrm{s}}_{xy} = \eta_{\mathrm{s}} V(t)/h$, being $V(t)$ is the probe's velocity (this expression can also be inferred by drawing an analogy with bulk simple shear flows). Thus, when $\nabla_{\mathrm{s}} \cdot \boldsymbol{\tau}^{\mathrm{s}}_{\mathrm{dev}} = \mathbf{0}$, bulk momentum

does not have an influence on $\boldsymbol{\tau}^{\rm s}_{\rm dev}$.

Nondimensionalizing Eq. 5 returns the following expression:

$$\left(\frac{\eta\, v}{l_{\rm bulk}}\right)\left(\tilde{\boldsymbol{\sigma}}^1 - \tilde{\boldsymbol{\sigma}}^2\right)\cdot \boldsymbol{n} = \left(\frac{\eta_{\rm s}\, v}{l_{\rm int}^2}\right)\left(\tilde{\nabla}_{\rm s}\cdot\tilde{\boldsymbol{\tau}}^{\rm s}\right), \tag{6}$$

where tildes represent dimensionless variables, v is a velocity scale for the interface, and $l_{\rm bulk}$ and $l_{\rm int}$ are the characteristic length scales over which bulk and interfacial momentum propagate, respectively[12,13]. For oscillatory flows, $\eta_{\rm s}$ is replaced by the complex viscosity $\eta_{\rm s}^*$ (as defined in Sec. 4.2.2 of the Tutorial).

We can then use Eq. 6 to define a shear Boussinesq number which, as in Eq. 13 of the Tutorial, reflects the ratio of bulk to interfacial stresses in a simple shear configuration:

$$\mathrm{Bq}_{\eta}^{(1)} = \frac{\tau^{\rm s}_{xy}}{\sigma^{\rm s}_{xy}\, l} = \frac{\eta_{\rm s}\, v/l_{\rm int}^2}{\eta\, v/l_{\rm bulk}}, \tag{7}$$

where $\tau^{\rm s}_{xy}$ and $\sigma^{\rm s}_{xy}$ indicate the relevant components of the surface and bulk stress tensors, and l is a relevant length scale. From Eq. 6, we see that

$$\tau^{\rm s}_{xy} \sim \eta_{\rm s}\, v/l_{\rm int}\;, \qquad \sigma^{\rm s}_{xy} \sim \eta\, v/l_{\rm bulk}\;, \qquad l \sim l_{\rm int}. \tag{8}$$

There are different choices for $l_{\rm bulk}$ and $l_{\rm int}$ which depend on the geometry, the flow kinematics, and the physical properties of the interface and bulk, as discussed in Fitzgibbon et al.[12].

For the bulk, $l_{\rm bulk}$ is the length scale over which bulk momentum propagates in the direction normal to the interface. For oscillatory deformations, there are two choices for $l_{\rm bulk}$:

$$l_{\rm bulk} = \begin{cases} (\eta/\rho\,\omega)^{1/2}, & \text{if } \; (\eta/\rho\,\omega)^{1/2} < H \\ H, & \text{if } \; (\eta/\rho\,\omega)^{1/2} \geq H \end{cases}, \tag{9}$$

where $(\eta/\rho\,\omega)^{1/2}$ is the Stokes boundary layer length and H is the depth of the liquid layer. For a water subphase and oscillation frequencies between 0.01 and 0.1 Hz, the Stokes boundary layer length lies between 3 and 10 mm. Typical PTFE troughs have a depth of 10 mm or less.

Conversely, l_{int} is the length scale over which interfacial momentum dissipates along the interface. There are three possible choices for l_{int}, as elegantly explained by in Fitzgibbon et al.[12]:

$$l_{\text{int}} = \begin{cases} l_{\text{bulk}}, & \text{if } \mathrm{Bq}_\eta^{(1)} \ll 1 \\ \sqrt{\eta_{\text{s}}\, l_{\text{bulk}}/\eta}, & \text{if } \mathrm{Bq}_\eta^{(1)} \approx 1 \\ h, & \text{if } \mathrm{Bq}_\eta^{(1)} \gg 1 \end{cases} \tag{10}$$

Which length scale should we use? The answer depends on the magnitude of η_{s} (or, equivalently, η_{s}^* for oscillatory flows)[12]:

- If η_{s} is really small, the motion of the interface will be governed by the motion of the bulk phases, and the length scale for bulk momentum transport will dictate the dynamics of the interface, such that $l_{\text{int}} \sim l_{\text{bulk}}$. [i]

- As η_{s} becomes larger, the motion of the interface will be governed by the interface's own material functions, and interfacial momentum will propagate beyond l_{bulk}. If η_{s} is still small, interfacial momentum transport will still be weak and the interfacial flow field will not "feel" the presence of the channel walls, resulting in complex, nonlinear velocity fields. In this case, $l_{\text{int}} \sim \sqrt{\eta_{\text{s}}\, l_{\text{bulk}}/\eta}$. This length scale is obtained by setting $\mathrm{Bq}_\eta^{(1)} = 1$ in Eq. 7.

- Let's now consider the specific geometries of the interfacial shear rheometers we use to conduct our measurements. Recall from Sec. 4.2.1 of the Tutorial, that the bicone,

[i] In most cases, using water as a subphase and at $\omega \geq 0.01$ Hz, $(\eta/\rho\,\omega)^{1/2} < h$, so bulk momentum will not propagate to the channel walls for oscillatory flows.

DWR, and ISR measurement probes are placed inside channels of width $2h$, as shown in Fig. 10e,g of the Tutorial for the DWR and ISR geometries. If η_{s} is large, interfacial momentum transport is strong and the interfacial flow will propagate all the way to the channel walls, and $l_{\mathrm{int}} \sim h$, the geometric length scale. Since interfacial momentum cannot propagate beyond h, the interfacial velocity and strain profiles will have simple analytical solutions in which $\nabla_{\mathrm{s}} \cdot \boldsymbol{\tau}^{\mathrm{s}}_{\mathrm{dev}} = \mathbf{0}$ (this is easier to visualize by drawing an analogy with bulk shear flows on i.e., double Couette geometries), as previously described, which affirms that bulk momentum does not influence τ^{s}_{xy}.

Note that the choice of l_{int} requires knowledge of η_{s} or η^*_{s}, which is a priori unknown. Thus, one must make sure that the choice of l_{int} and the resulting value of $\mathrm{Bq}^{(1)}_{\eta}$ are mutually consistent.

Thus, only when η_{s} is large and $l_{\mathrm{int}} \sim h$ will bulk stresses not influence the interfacial stress. Let's now express this condition in a more mathematical and quantitative form. We can affirm that, in any case, $l_{\mathrm{int}} \leq h$. Thus, a conservative and practical choice for l_{bulk} and l_{int} are the geometric length scales H and h, respectively. This allows us to define a conservative estimate for $\mathrm{Bq}^{(1)}_{\eta}$ in Eq. 7:

$$\mathrm{Bq}^{(1)}_{\eta} = \frac{\eta_{\mathrm{s}}\, v/l^2_{\mathrm{int}}}{\eta\, v/l_{\mathrm{bulk}}} = \frac{\eta_{\mathrm{s}}\, H}{\eta\, h^2}. \tag{11}$$

Therefore, $\mathrm{Bq}^{(1)}_{\eta} \gg 1$ for bulk stresses to not influence the interfacial shear flow profile and for τ^{s}_{xy} to be independent of bulk momentum transport.

3.2 The influence of bulk hydrodynamics on the motion of the probe

Rotational and channel-based interfacial shear rheometers described in the main manuscript (i.e., bicone, DWR, and ISR) use a probe in contact with the interface to impose an interfacial shear deformation. The raw data acquired by these rheometers is some measurement of the

force applied on the probe (torque applied by the rotor in the DWR, or magnetic field gradient in the ISR) and some measurement of the imposed deformation (angular displacement of the DWR or position of the needle in the ISR)[13–18]. The bulk and interfacial stresses must therefore be obtained from the probe's motion.

Setting up a force balance equation for the probe reveals that the probe's inertia is equal to the forces acting on it:

$$M\frac{dV(t)}{dt} = F_{\mathrm{bulk}} + F_{\mathrm{int.}} + F_{\mathrm{appl.}}, \tag{12}$$

where M is the probe's mass and $V(t)$ is its velocity, which is itself a function of time for oscillatory flows. The probes are subjected to three forces: (1) drag forces from the bulk, F_{bulk}, (2) drag forces from the interface, $F_{\mathrm{int.}}$, and (3) forces that are applied by the instrument in order to drive probe motion, $F_{\mathrm{appl.}}$. To neglect bulk contributions, $F_{\mathrm{int.}} \gg F_{\mathrm{bulk}}$. A Boussinesq number can be defined as the ratio of the interfacial to bulk forces on the probe:

$$\mathrm{Bq}_{\eta}^{(2)} = \frac{F_{\mathrm{int.}}}{F_{\mathrm{bulk}}} = \frac{\tau^{\mathrm{s}}_{xy}\, P_{\mathrm{p}}}{\sigma^{\mathrm{s}}_{xy}\, A_{\mathrm{p}}} = \frac{(\eta_{\mathrm{s}}\, V/l_{\mathrm{int}})\; P_{\mathrm{p}}}{(\eta\, V/l_{\mathrm{bulk}})\; A_{\mathrm{p}}} = \frac{\eta_s}{\eta\, a}\frac{l_{\mathrm{bulk}}}{l_{\mathrm{int}}}, \tag{13}$$

where τ^{s}_{xy} and σ^{s}_{xy} indicate the relevant components of the surface and bulk stress tensors, respectively. Since the probe has a finite size, its contact with the interface takes place along the probe perimeter, P_{p}. Additionally, its contact with the bulk phase (let's only consider the more viscous of the two bulk phases) takes place over the probe's surface area, A_{p}. Thus, a is the ratio of the contact surface area to the contact line perimeter, and for the DWR and ISR geometries, a is equal to the probe radius. Most authors assume that $l_{\mathrm{bulk}} \sim l_{\mathrm{int}}$, such that

$$\mathrm{Bq}_{\eta}^{(2)} = \frac{\eta_s}{\eta\, a}, \tag{14}$$

although we have previously seen that this assumption is not necessarily true.

If $\mathrm{Bq}_\eta^{(2)} \gg 1$, the influence of the interface on the motion of the probe is much more important than that of the bulk. Therefore, the physical model used to analyze our experimental data (i.e., the probe motion) can neglect the drag from the bulk. However, if $\mathrm{Bq}_\eta^{(2)} \sim 1$, we must be extremely careful to use a physical model that accounts for the drag from the bulk. What if $\mathrm{Bq}_\eta^{(2)} \ll 1$? In this scenario, the physical quantity we are trying to calculate (η_s or η_s^*) has very little weight in the raw data we are acquiring with the rheometer, which means that the relative error in our result can be quite large, and the measurement will lie outside the instrument's operating windows[19,20] (see Renggli et al.[20] and Guzmán et al.[21] for more information on operating ranges for different interfacial shear rheometers).

4 Drop/bubble-based methods and the Young-Laplace equation

The Young-Laplace equation is obtained from the dot product between the interfacial momentum balance (Eq. 4 in the Tutorial) and $\boldsymbol{n}$, and assumes that (1) the bulk fluids are stationary (i.e., $\boldsymbol{\sigma}^1 = -P^1\boldsymbol{I}; \quad \boldsymbol{\sigma}^2 = -P^2\boldsymbol{I}$), (2) there are no rheological stresses (i.e., $\boldsymbol{\tau}^\mathrm{s} = \boldsymbol{0}$), and (3) $\sigma_{\alpha\beta}$ is spatially homogeneous:

$$P^2 - P^1 = \sigma_{\alpha\beta} \left(\nabla_\mathrm{s} \cdot \boldsymbol{n} \right). \tag{15}$$

Eq. 15 describes the balance between pressure and surface forces (remember from 2.5 in the Tutorial that capillary stresses induce a pressure jump across curved interfaces). Images of the drop/bubble are recorded with a camera, and the interface contour is extracted via image processing algorithms and used in Eq. 15 to calculate $\sigma_{\alpha\beta}$ and changes thereof[7,22–24].

In ADSA, the drop/bubble must be large enough to be deformed by gravity, as quantified by the Bond or Worthington numbers[22,23,25]. Such pendant configurations make it possible to calculate ΔP directly from the drop geometry[22]. Straightforward shape-fitting

techniques are widely available, either as part of commercial tensiometers or as open-source routines[22,23,26]. CPT is a common alternative to ADSA, which uses much smaller, sub-hemispherical drops/bubbles in which hydrostatic pressure effects are negligible[25,27,28]. Thus, the interface adopts a spherical shape of constant curvature, obtained by fitting a circle to its contour[27,29]. Although image analysis is vastly simplified in CPT, ΔP must be obtained using a pressure transducer. Remember that Eq. 15 is only valid when the interfacial stress is isotropic, which means that these methods can **only** be employed for (1) simple interfaces without a rheological response or (2) unstrained complex interfaces, where a rheological response has not yet been elicited (remember that rheological stresses arise whenever the interface is deformed relative to its reference configuration; thus, if a complex interface has not yet been deformed, no rheological stresses have accumulated). Drop-based methods can also be used to characterize complex interfaces, albeit with certain modifications. For more information on drop/bubble *rheometry*, refer to Danov et al.[24], Nagel et al.[23], Carvajal et al.[30], and Rodríguez-Hakim et al.[26].

References

(1) Thiel, C.; Voss, J.; Martin, R. J.; Neff, P. Shear, pure and simple. *International Journal of Non-Linear Mechanics* **2019**, *112*, 57–72.

(2) Ogden, R. W. *Nonlinear Elastic Deformations*; John Wiley & Sons Ltd., 1984.

(3) Tein, Y.; Thompson, B. R.; Majkrzak, C.; Maranville, B.; Renggli, D.; Vermant, J.; Wagner, N. J. Instrument for measurement of interfacial structure-property relationships with decoupled interfacial shear and dilatational flow: "Quadrotrough". *Review of Scientific Instruments* **2022**, *93*.

(4) Ashkenazi, D.; Pham, K.; Vermant, J.; Wagner, N. J.; Gottlieb, M. Evaluation of a novel multimode interfacial rheometer. *Journal of Rheology* **2024**, *68*, 785–799.

(5) Jaensson, N.; Vermant, J. Tensiometry and rheology of complex interfaces. *Current opinion in colloid & interface science* **2018**, *37*, 136–150.

(6) Verwijlen, T.; Leiske, D.; Moldenaers, P.; Vermant, J.; Fuller, G. Extensional rheometry at interfaces: Analysis of the Cambridge Interfacial Tensiometer. *Journal of Rheology* **2012**, *56*.

(7) Deen, W. M. *Analysis of transport phenomena*; Oxford University Press, 1998.

(8) Leal, L. G. *Advanced transport phenomena: fluid mechanics and convective transport processes*; Cambridge university press, 2007; Vol. 7.

(9) Horgan, C.; Murphy, J. Simple shearing of incompressible and slightly compressible isotropic nonlinearly elastic materials. *Journal of Elasticity* **2010**, *98*, 205–221.

(10) Shrivastava, S.; Ghosh, C.; Jonas, J. J. A comparison of the von Mises and Hencky equivalent strains for use in simple shear experiments. *Philosophical Magazine* **2012**, *92*, 779–786.

(11) Verwijlen, T.; Imperiali, L.; Vermant, J. Separating viscoelastic and compressibility contributions in pressure-area isotherm measurements. *Advances in colloid and interface science* **2014**, *206*, 428–436.

(12) Fitzgibbon, S.; Shaqfeh, E. S.; Fuller, G. G.; Walker, T. W. Scaling analysis and mathematical theory of the interfacial stress rheometer. *Journal of Rheology* **2014**, *58*, 999–1038.

(13) Sánchez-Puga, P.; Tajuelo, J.; Pastor, J. M.; Rubio, M. A. Flow field-based data analysis in interfacial shear rheometry. *Advances in Colloid and Interface Science* **2021**, *288*, 102332.

(14) Tajuelo, J.; Pastor, J.; Martínez-Pedrero, F.; Vázquez, M.; Ortega, F.; Rubio, R.;

Rubio, M. Magnetic microwire probes for the magnetic rod interfacial stress rheometer. *Langmuir* **2015**, *31*, 1410–1420.

(15) Verwijlen, T.; Moldenaers, P.; Stone, H. A.; Vermant, J. Study of the flow field in the magnetic rod interfacial stress rheometer. *Langmuir* **2011**, *27*, 9345–9358.

(16) Brooks, C. F.; Fuller, G. G.; Frank, C. W.; Robertson, C. R. An interfacial stress rheometer to study rheological transitions in monolayers at the air- water interface. *Langmuir* **1999**, *15*, 2450–2459.

(17) Reynaert, S.; Brooks, C. F.; Moldenaers, P.; Vermant, J.; Fuller, G. G. Analysis of the magnetic rod interfacial stress rheometer. *Journal of Rheology* **2008**, *52*, 261–285.

(18) Vandebril, S.; Vermant, J.; Moldenaers, P. Efficiently suppressing coalescence in polymer blends using nanoparticles: role of interfacial rheology. *Soft Matter* **2010**, *6*, 3353–3362.

(19) Tajuelo, J.; Pastor, J.; Rubio, M. A. A magnetic rod interfacial shear rheometer driven by a mobile magnetic trap. *Journal of Rheology* **2016**, *60*, 1095–1113.

(20) Renggli, D.; Alicke, A.; Ewoldt, R. H.; Vermant, J. Operating windows for oscillatory interfacial shear rheology. *Journal of Rheology* **2020**, *64*, 141–160.

(21) Guzmán, E.; Tajuelo, J.; Pastor, J. M.; Rubio, M. Á.; Ortega, F.; Rubio, R. G. Shear rheology of fluid interfaces: Closing the gap between macro-and micro-rheology. *Current opinion in Colloid & interface science* **2018**, *37*, 33–48.

(22) Rotenberg, Y.; Boruvka, L.; Neumann, A. Determination of surface tension and contact angle from the shapes of axisymmetric fluid interfaces. *Journal of colloid and interface science* **1983**, *93*, 169–183.

(23) Nagel, M.; Tervoort, T. A.; Vermant, J. From drop-shape analysis to stress-fitting elastometry. *Advances in Colloid and Interface Science* **2017**, *247*, 33–51.

(24) Danov, K. D.; Stanimirova, R. D.; Kralchevsky, P. A.; Marinova, K. G.; Alexandrov, N. A.; Stoyanov, S. D.; Blijdenstein, T. B.; Pelan, E. G. Capillary meniscus dynamometry - Method for determining the surface tension of drops and bubbles with isotropic and anisotropic surface stress distributions. *Journal of Colloid and Interface Science* **2015**, *440*, 168–178.

(25) Reichert, M. D.; Alvarez, N. J.; Brooks, C. F.; Grillet, A. M.; Mondy, L. A.; Anna, S. L.; Walker, L. M. The importance of experimental design on measurement of dynamic interfacial tension and interfacial rheology in diffusion-limited surfactant systems. *Colloids and Surfaces A: Physicochemical and Engineering Aspects* **2015**, *467*, 135–142.

(26) Rodríguez-Hakim, M.; Jaensson, N.; Vermant, J. Towards operating windows for pendant drop methods: tensiometry and rheometry of elastic interfaces. *Rheologica Acta* **2025**, 1–21.

(27) Kotula, A. P.; Anna, S. L. Regular perturbation analysis of small amplitude oscillatory dilatation of an interface in a capillary pressure tensiometer. *Journal of Rheology* **2015**, *59*, 85–117.

(28) Ravera, F.; Loglio, G.; Kovalchuk, V. I. Interfacial dilational rheology by oscillating bubble/drop methods. *Current Opinion in Colloid & Interface Science* **2010**, *15*, 217–228.

(29) Iasella, S. V.; Barman, S.; Ciutara, C.; Huang, B.; Davidson, M. L.; Zasadzinski, J. A. Microtensiometer for Confocal Microscopy Visualization of Dynamic Interfaces. *Journal of visualized experiments : JoVE* **2022**,

(30) Carvajal, D.; Laprade, E. J.; Henderson, K. J.; Shull, K. R. Mechanics of pendant drops and axisymmetric membranes. *Soft Matter* **2011**, *7*, 10508–10519.
